# The Potential Role of Curcumin in Modulating the Master Antioxidant Pathway in Diabetic Hypoxia-Induced Complications

**DOI:** 10.3390/molecules26247658

**Published:** 2021-12-17

**Authors:** Somayyeh Ghareghomi, Mahdie Rahban, Zainab Moosavi-Movahedi, Mehran Habibi-Rezaei, Luciano Saso, Ali Akbar Moosavi-Movahedi

**Affiliations:** 1 Institute of Biochemistry and Biophysics, University of Tehran, Tehran 1417466191, Iran; ghareghomi.s@ut.ac.ir (S.G.); mrohban@ut.ac.ir (M.R.); 2 Chemistry and Chemical Engineering Research Center of Iran, Tehran 1496813151, Iran; z.moosavi@ccerci.ac.ir; 3 School of Biology, College of Science, University of Tehran, Tehran 1417466191, Iran; 4 Center of Excellence in NanoBiomedicine, University of Tehran, Tehran 1417466191, Iran; 5 Department of Physiology and Pharmacology “Vittorio Erspamer,” Sapienza University of Rome, 00185 Rome, Italy; luciano.saso@uniroma1.it; 6 UNESCO Chair on Interdisciplinary Research in Diabetes, University of Tehran, Tehran 1417466191, Iran

**Keywords:** Keap1-Nrf2, diabetes, oxidative stress, curcumin, antioxidant enzymes, hypoxia-inducible factor 1 (HIF-1), catalase

## Abstract

Oxidative stress is the leading player in the onset and development of various diseases. The Keap1-Nrf2 pathway is a pivotal antioxidant system that preserves the cells’ redox balance. It decreases inflammation in which the nuclear trans-localization of Nrf2 as a transcription factor promotes various antioxidant responses in cells. Through some other directions and regulatory proteins, this pathway plays a fundamental role in preventing several diseases and reducing their complications. Regulation of the Nrf2 pathway occurs on transcriptional and post-transcriptional levels, and these regulations play a significant role in its activity. There is a subtle correlation between the Nrf2 pathway and the pivotal signaling pathways, including PI3 kinase/AKT/mTOR, NF-κB and HIF-1 factors. This demonstrates its role in the development of various diseases. Curcumin is a yellow polyphenolic compound from *Curcuma longa* with multiple bioactivities, including antioxidant, anti-inflammatory, anti-tumor, and anti-viral activities. Since hyperglycemia and increased reactive oxygen species (ROS) are the leading causes of common diabetic complications, reducing the generation of ROS can be a fundamental approach to dealing with these complications. Curcumin can be considered a potential treatment option by creating an efficient therapeutic to counteract ROS and reduce its detrimental effects. This review discusses Nrf2 pathway regulation at different levels and its correlation with other important pathways and proteins in the cell involved in the progression of diabetic complications and targeting these pathways by curcumin.

## 1. Introduction

The cellular redox homeostasis system protects cells from oxidative attacks of endogenous and exogenous sources of ROS and RNS. ROS is produced endogenously by the respiratory chain of mitochondria or enzymatic reactions of cyclooxygenases, NAD(P)H oxidase, lipoxygenase, and xanthine oxidase in peroxisomes or endoplasmic reticulum [1,2]. Besides, ROS is exogenously produced by ultraviolet and ionizing radiation and the presence of toxicants or pathologic insults [3]. Oxidative stress and inflammation are the primary mediators for the onset and development of various diseases [4]. Therefore, dealing with stress conditions in cells is a fundamental mechanism for preventing diseases and their complications. Oxidative stress is unbalanced conditions between ROS generation and impaired antioxidants valence [5,6,7,8,9]. Overproduction of ROS leads to cell damage and disruption of fundamental cell processes through structural and functional modifications of cell proteins, nucleic acids, and lipids.

Hence, cells develop non-enzymatic and enzymatic antioxidant systems to protect themselves from inflammation and pathogenesis of a broad range of oxidative stress-related diseases [10,11]. ROS is neutralized via thiol-based molecules like GSH and thioredoxin (Trx) or even by serum albumin as a non-enzymatic antioxidant system, as well as balanced by the enzymatic antioxidant system [3,12].

Some studies have shown decreased antioxidant response in cells leads to various diseases, including diabetes, cancer, and AD. Among these diseases, diabetes and its complications have a high prevalence today. Based on the International Diabetes Federation (IDF) reports, diabetes is spreading rapidly and is expected to overstep 640 million by 2040. Diabetes complications are observed in both T1DM and T2DMas the leading reasons for death in diabetic patients [13]. Diabetes is associated with hyperglycemia, glucotoxicity, and oxidative stress, which collectively develop AGEs lipid peroxidation products toward intensifying ROS generation in the cell [14,15]. Therefore, exogenous and endogenous ROS and electrophiles induce the cytosolic two Keap1 and one Nrf2 complex toward nuclear translocation of Nrf2 where it binds to antioxidant response elements after making complex with sMAF protein (Nrf2-sMaf) to trigger transcription of cytoprotective genes of phase II enzymes [16,17,18,19,20]. In Nrf2/Keap1/ARE pathway inducers modify the specific reactive cysteine residues of Keap1 that results in Keap1conformational change followed by Nrf2 translocation to the nucleus. Furthermore, nuclear localization of Nrf2 regulated through phosphorylation of some of its threonine or serine residues by various kinases such as mitogen-activated protein kinases (MAPKs), PI3K/AKT, and PKC [21].

Curcumin is a natural polyphenol component obtained from the rhizome of the plant *Curcuma longa* and utilized for a long time in traditional medicines [22]. A large body of findings indicated that this molecule demonstrates potential therapeutic properties in combating various inflammatory diseases and cancer [23,24,25]. According to our studies, catalase, the oldest antioxidant enzyme discovered, can be activated by curcumin by 180% [26]. Considering this information, we overview Keap1-Nrf2 pathway and the role of curcumin in Nrf2 related pathways in this review. Moreover, the therapeutic applications of curcumin in reducing diabetes and its complications are discussed.

## 2. Molecular Regulation of the Keap1-Nrf2 Signaling Pathway

**Figure 1:**
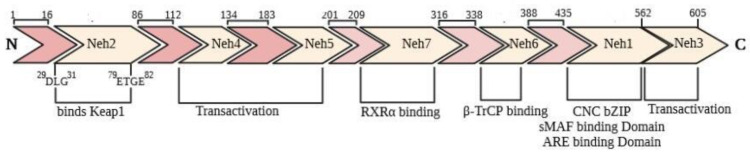
Domain structure of Nrf2. The seven functional domains (Neh1–7) and their positions.

Nrf2 is commonly present in every part and especially in the organs involved in detoxification processes and metabolism. The Keap1–Nrf2 pathway plays a vital role in protecting cells against endogenous and exogenous oxidative stresses damages. As depicted in Figure 1, Nrf2 has 605 amino acids residues that were arranged in 7 functional domains identified as Neh domains (Neh1–Neh7) [27]. This protein is a bZIP (basic-region leucine zipper) transcription factor and belongs to the Cap ‘n’ collar (CNC) family of regulatory proteins. Neh1 consists of a CNC essential section for DNA binding and has a leucine zipper segment for hetero-dimerization with sMAF [28]. Neh2 is involved in the inactivation of Nrf2 through binding to Keap1 in the cytoplasm. Nrf2 binding to Keap1 occurs by two separate motifs of Nrf2, the ETGE, and DLG motifs. Therefore, two molecules of Keap1 via one molecule of Nrf2 (2:1) are present in inactive forms of the Keap1-Nrf2 complex. Seven lysine residues between DLG and ETGE motifs of Nrf2 are essential for its ubiquitination and degradation. Neh3, Neh4, and Neh5 act as transactivation domains. The transactivation domains of Neh3, Neh4, and Neh5 mediates Nrf2 interaction with other coactivators. Neh6 domain with serine-rich residues mediates Nrf2 degradation in the nucleus [29]. Finally, amino acids 209–316 are described as the Neh7 domain binds to the retinoid X receptor α (RXRα), to inhibit the Nrf2-ARE signaling pathway [30]

Keap1 is a zinc-metalloprotein that consists of 624 amino acid residues and belongs to the BTB-Kelch family of proteins. It has five structural regions: NTR, BTB domain, intervening region (IVR), Kelch domain (KD), and C-terminal region (CTR). The KD with a 6-bladed β-propeller conformation is responsible for Keap1 binding to the DLG and ETGE motifs in Neh2 domain of Nrf2 via the “hinge and latch” model. Modifying cysteine residues of C273 and C288 in the IVR region and C151 in the BTB domain as targets of oxidants and electrophiles and leads to conformational changes in the Keap1 that causes Nrf2 detachment. Also, specific toxins appear to modify C226, C434, and C613 residues [31]. In the nucleus, the Nrf2-sMaf heterodimer regulates ARE, resulting in the expression of a list of antioxidant enzymes. The functional activity of Nrf2 is determined through its expression level and its nuclear localization.

Under normal non-stress conditions, Nrf2 degrades by the ubiquitin-proteasome system, and its half-life is about 20–30 min [32]. Keap1, as a multifunctional molecule, can bind to Nrf2 and promotes its ubiquitin-mediated degradation. The Kelch domain of the Keap1 binds to the ETGE motif of the Neh2 domain of Nrf2 through a high affinity (as a hinge) and low-affinity to DLG motifs (as latch). Keap1-Nrf2 ubiquitination is mediated by a region of 7 lysine residues of the Keap1-cullin-RING-based BCR E3 ubiquitin-protein ligase complex, which mediate the ubiquitination and subsequent 26S proteasomal degradation of Nrf2. Degradation of Nrf2 maintains the expression of its target genes in basal conditions [33].

### 2.1. Kinase-Dependent Regulations of Nrf2

**Figure 2:**
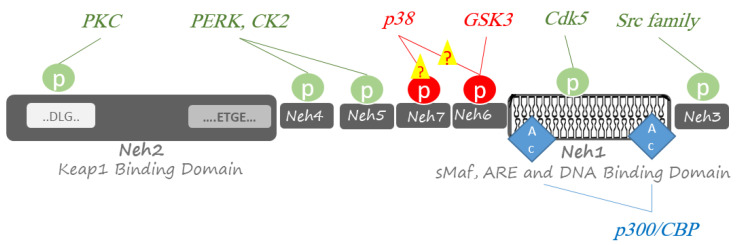
Post-translational phosphorylation and acetylation of Nrf2. Phosphorylation of Nrf2 is performed at Ser/Thr residues by multiple protein kinases to activate Nrf2-related responses to oxidative stresses (green circles) or proteasomal degradation by a Cullin1/Rbx1 complex (red circles). Acetylation of Nrf2 at several Lys residues by transcriptional co-activators p300 and CREB-binding protein (CBP); p300/CBP has a boosting effect on Nrf2 for binding DNA (blue diamond).

In addition to the primary Keap1 dependent mechanism, some studies showed that Nrf2 post-translational modification, including phosphorylation and acetylation, is involved in its regulation [34]. Among potentially 450 kinases in the human genome [35], several kinases have been reported to play fundamental roles in positively or negatively regulating Nrf2 (Figure 2). Some kinases have a limited function, whereas others are widely active to phosphorylate many proteins. In continuing, the kinases that mediate in signal transduction at ARE genes through phosphorylation and modulation of Nrf2 are discussed.

#### 2.1.1. Protein kinase RNA-Like Endoplasmic Reticulum Kinase (PERK)

PERK is a protein kinase that belongs to eIF2α kinase subfamily. PERK is composed of the cytoplasmic and kinase domains. Activation and autophosphorylation of these domains occur after stimulation and it can sense stress and conduct ER stress signals [36]. Malfunctions of the endoplasmic reticulum (ER) are triggered by multiple factors that could result in unfolded proteins response (UPR), resulting in ER stress contributing to ROS-mediated cell apoptosis. In response, PERK, a Ser/Thr protein kinase that accounts as an ER stress sensor [37], phosphorylate Neh4 domain of Nrf2 and induce disintegration of Nrf2-Keap1 complex to promote Nrf2 nuclear translocation. Moreover, Zhong-Wei Liu et al. have reported PERK’s possible contribution in ROS-induced ER stress-mediated apoptosis in diabetic cardiomyopathy (DCM) [38].

#### 2.1.2. Protein Kinase C (PKC)

Protein kinase C signifies a family of serine/threonine kinases that belong to the AGC superfamily of protein kinases. PKCs have a conserved kinase domain coupled to a series of differentially activated regulatory domains [39]. The PKC family is centrally involved in the spatial control of signal transduction in cells. It has been hypothesized that PKC as a Ser/Thr kinase directly phosphorylates Ser40 in Neh2 domain of Nrf2 to activate Nrf2-related responses to oxidative stresses [40].

#### 2.1.3. Casein Kinase 2 (CK2)

CK2 is a small family of closely related protein kinases that have a much wider specificity and are expected to phosphorylate hundreds of diverse proteins within cells. it most often seems to exist in tetrameric complexes consisting of two catalytic subunits and two regulatory subunits [41]. Phosphorylation of specific residues in Neh4 and Neh5 domains of Nrf2 by CK2as a Ser/Thr protein kinase leads to the nuclear translocation of Nrf2 in neuroblastoma cells [42,43]. In addition, CK2 downregulation inhibits Nrf2 translocation in breast cancer by deactivating AMPK [42,44].

#### 2.1.4. Src Family of Tyrosine Protein Kinases

Src family tyrosine-protein kinases, including Src and Lyn subfamilies, have an essential role in the phosphorylation of various proteins involved in the fundamental cell processes [45]. While Nrf2 phosphorylation on tyrosine 568 by Src subfamily leads to its proteasomal degradation [46,47,48,49], Fyn as the Lyn subfamily regulates the Nrf2 pathway through its nuclear export.

#### 2.1.5. Cyclin-Dependent Kinase 5 (Cdk5)

CDK5 is a proline-directed serine/threonine kinase belonging to the family of cyclin-dependent kinases. It needs association with a regulatory partner, p35 and p39 for kinase activation [50] Jimenez-Blasco et al. demonstrated that the Cdk5-Nrf2 signaling pathway seems to be ubiquitous in astrocytes against oxidants [51]. The complex of p35/Cdk5 phosphorylates Nrf2 at Thr395, Ser433, and Thr439 to promote Nrf2 translocation to the nucleus and boosts the expression of GSH genes [51]. 

#### 2.1.6. Mitogen-Activated Protein Kinase (MAPK)

MAPKs are classified into three groups: the JNK, the p38 MAP kinases, and the ERK [52]. JNK and p38 MAPK are SAPK activated by stress stimuli [53]. The SAPKs family consist of three isoforms of JNK (1, 2 and 3) and four isoforms of p38(α, β, δ and γ) [54]. Various stimuli such as toxins, cytokines, drugs, environmental stresses, and metabolic disorders can activate JNKs. Activation of JNKs occurs following its phosphorylation on tyrosine and threonine in the preserved Thr-Pro-Tyr motif in their activation loop [54]. There are over 50 proteins as JNK substrate such as c-Jun, insulin IRS-1, c-myc, p53, and various transcriptional factors. Also, activation of p38 MAPK in response to multiple stimuli occurs through phosphorylation in the activation loop sequence Thr-Gly-Tyr [55,56,57]. One of the most basic pathways in Nrf2 system regulation is MAPK signaling. Activation of these kinases in response to stresses has a delicate correlation to the Nrf2-related pathway. Phosphorylation of Nrf2 by p38 leads to the formation of Nrf2-Keap1copmplex and Nrf2 pathway downregulation [58,59]. In contrast, the kinase activity of JNKs on Nrf2 promotes its nuclear translocation and activation of Nrf2 pathway [60].

#### 2.1.7. Phosphatidylinositol 3-Kinase (PI3K)

PI3K is an upstream kinase involved in AKT/mTOR signaling, and its activation through various stimuli is responsible for triggering other kinases in this pathway [61,62]. There are three classes of phosphatidyl inositol 3-kinase (PI3Ks) with various subunits. Stimulation of growth factor receptor (GFR) leads to phosphorylation phosphatidyl inositol diphosphate (PIP2) and produced PIP3 by PI3K. Then PIP3 activated protein kinase B (Akt) by phosphorylation on T308 and S437 [63]. Activation of these kinases is responsible for triggering other kinases in this pathway. Numerous studies have shown PI3 kinase/AKT signaling is a regulator pathway of the Nrf2 system [64,65]

#### 2.1.8. Glycogen Synthase Kinase-3 (GSK3)

GSK3, a Ser/Thr protein kinase in eukaryotes, exists in GSK3α and GSK3β forms, with a highly preserved catalytic domain. GSK3β is a fundamental regulator of various metabolic processes and cell signaling pathways [66]. It phosphorylates some serine residues in the Neh6 domain of the Nrf2 to form a degradation domain that is bound to β-TrCP and characterized for proteasome degradation by a Cullin1/Rbx1 complex [67,68,69]. However, it is thought that this signaling is not related to the redox state of the cells [34,70].

### 2.2. Other Factors Involved in the Regulation of Nrf2

In addition to ubiquitination and phosphorylation, acetylation also has a pivotal role in Nrf2 pathway regulation [71]. Histone acetylation by transcriptional co-activators p300 and cAMP-response element-binding protein CBP is the primary step in transcription. Under oxidative stress conditions, p300/CBP binds to Nrf2 and acetylates several lysine residues within the Neh1 domain of Nrf2 [72]. Neh1 domain has a DNA binding section, and its lysine modification has a boosting efficacy on Nrf2 for binding DNA.

Nrf2 and NF-κB pathways have a fundamental role in response to oxidative stress and inflammation. NF-κB as a nuclear transcription factor participates in the cellular response to various stimuli such as types of stress, cytokines, ROS, and free radicals. NF-κB complex consists of a set of structurally similar proteins of the Rel family and that are regulated through transfers between cytoplasm and nucleus in response to different stimulation [73]. There are five protein members of the NF-κB family involving RelA (p65), c-Rel, RelB, p50, and p52. Based on some studies, RelA has a negative role in ARE-linked gene expression [74,75]. Since a variety of anti-inflammatory or anti-carcinogenetic phytochemicals suppress NF-κB signaling and activate the Nrf2-ARE pathway as well. Some findings have revealed that RelA overexpression causes downregulation or even elimination of the Nrf2 pathway. RelA boosts the employment of histone deacetylase 3 (HDAC3) to associate with either CBP or sMAF, preceding to local histone hypoacetylation and preventing heterodimer formation with Nrf2, therefore downregulation of Nrf2-related gene expression [76]. Also, Nrf2 and RelA tend to bind transcriptional co-activators CBP, but RelA has a stronger affinity than Nrf2 for CBP. Therefore, the RelA-CBP complex formation is more stable as compared to the Nrf2-CBP complex. On the other hand, when NF-κB is activated, cytokine levels and interleukins increase ROS production and Nrf2-related gene expression. At the same time, the Nrf2 pathway can modify the anti-inflammatory pathways by the inhibition of the NF-κB pathway [76].

MicroRNAs (miRNAs) can modulate oxidative stress conditions in cells by regulating the Nrf2 pathway. In recent years, studies have focused on the roles of miRNAs in regulating the activity of several proteins, including Nrf2. miRNAs involved in regulating the Nrf2 pathway include miR-144, miR-28, miR-34, and miR-200 [77,78,79,80]. An increase in miR-144 expression decrease Nrf2 protein levels and reduces GSH regeneration in erythrocytes of homozygous sickle cell disease (SCD) [79]. MiR-28 has been displayed to regulate the Nrf2 pathway by degrading Nrf2 mRNA and protein in breast epithelial cells [80]. Whereas miR-200 can regulate the Nrf2 pathway by Keap1 mRNA targeting and miR-34, regulate the Nrf2 pathway and a set of downstream genes in response to oxidative stress in HEK 293 cells [78].

**Figure 3:**
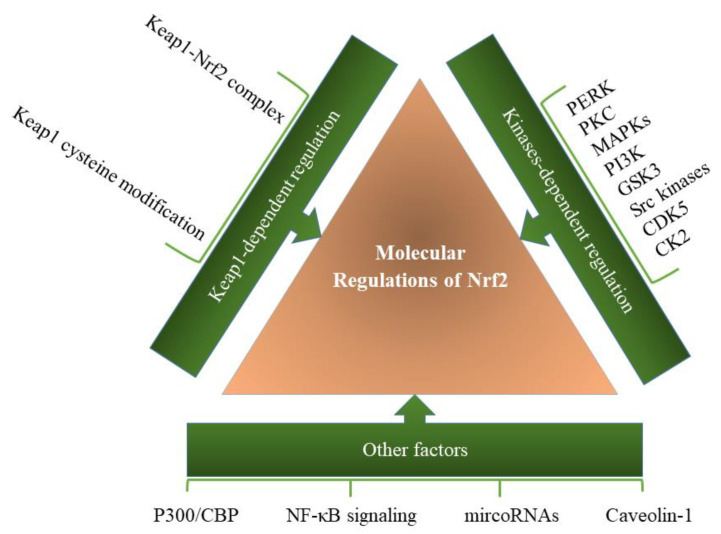
Molecular regulation of the Keap1-Nrf2 pathway. A variety of factors and pathways are involved in regulating the function of the Keap1-Nrf2 pathway, which can be modulated through various factors in the cell.

Another important Nrf2 partner is caveolin-1 (Cav-1), a scaffold protein in caveolae membranes, which transmits various signals and the uptake of lipophilic agents. Cav-1 regulates multiple critical biological processes through interaction with different proteins, including Nrf2. Based on Zheng et al.’s study, Cav-1 directly interacts with Nrf2, resulting in suppression of antioxidant enzymes expression [81]. Specific molecular regulations of Keap1-Nrf2 pathway are summarized in Figure 3.

## 3. Keap1/Nrf2/ARE Pathway and Antioxidant Enzyme Regulation

The cytoprotective Nrf2/Keap1 pathway is a key player in the prevention of oxidative stress [82]; therefore, its dis-regulation results in diabetes and its complications as a characteristic of insulin resistance (IR) [83]. Nrf2 is inactive while being bound to Keap1 in the cytoplasm, in which Keap1 restricts nuclear translocation of Nrf2 and presents it to proteasomal degradation [18]. However, Nrf2 nuclear translocation of Nrf2 switches on the gene transcription of cellular antioxidant enzymes to scavenge free radicals and prevent oxidative stress consequences, more importantly, diabetes and its complications. Therefore, activation of Keap1-Nrf2 pathway and antioxidant enzymes create a protective role against diabetic complications [84]. Cells developed non-enzymatic and enzymatic antioxidant systems to protect themselves from cell inflammation and pathogenesis of many oxidative stress diseases [10,11]. ROS is directly neutralized via thiol-based small molecules such as GSH and Trx as a non-enzymatic antioxidant system and balanced by enzymatic antioxidant systems [3]. Nrf2 related response to oxidative stress in cells involves increasing the expression and production of several antioxidant enzymes. The downstream enzymes as targets of the Keap1/Nrf2/ARE pathway have expressed under oxidative stress to role efficiently in keeping the oxidative balance of the cells have listed in Table 1, and some of them are discussed in detail.

### 3.1. Heme Oxygenase-1

The heme oxygenase-1 (HO-1; EC 1.14.14.18), which detoxifies heme molecules, is introduced by Nrf2 target genes [103,104]. Heme oxygenases are encoded by HMOX genes that are ubiquitous in most living organisms, and it seems that this enzymatic reaction has appeared early during evolution [105]. Its deficiency causes cytotoxicity with increasing H_2_O_2_ and hemin. HO-1 catalyzes the rate-limiting step in heme catabolism to generate biliverdin, free iron and carbon monoxide (CO) as a breakdown product by degradation of the heme molecule. HO-1 Biliverdin reductase converts biliverdin to bilirubin, which directly neutralizes superoxide, hydroxyl, and peroxy-nitrite radicals by direct interaction with bilirubin. Therefore, HO-1 protects the cell from oxidative stress during inflammatory stress in obesity and diabetes [106,107]. HO-1 up-regulation can increase insulin secretion, thereby reducing hyperglycemia. Obesity-mediated development of hyperglycemia has a direct effect on HO-1 suppression [108]. *HMOX1* is transcriptionally up-regulated by a variety of signal transduction pathways that activate different transcription factors. ROS can draw out heme releasing from hemoproteins and generate oxidative stress. The HMOX1 promoter consists of multiple DNA-responsive elements, which are triggered by specific transcription factors. Nrf2/sMAF heterodimers induced by ROS bind to stress-responsive elements (StREs) in the HMOX1 promoter and promote OH-1 production [105]. 

### 3.2. NAD[P]H Quinone Dehydrogenase-1

The NAD(P)H quinone dehydrogenase-1 (NQO-1; EC 1.6.5.2) is a downstream target of Nrf2 in protection against diabetes and metabolic disorders. In addition, in high-fat diets of mice, Nrf2 activation and NQO-1 overexpression could improve glucose and insulin metabolism [109]. NQO1 is a two-electron donor and detoxifies quinones and their derivatives and protects cells against oxidative stress. NQO1 catalyzes the reduction of quinones to hydroquinones, is a cytosolic homo-dimeric flavoprotein, and utilizes either NADH or NADPH as a reducing cofactor [85]. NQO-1 consists of a FAD prosthetic group, which is essential for the stability and function of the enzyme. When NQO1 is expressed at very high levels and SOD levels are low, NQO1-mediated reduction of superoxide. This ability depends on the generation of FADH2 following enzyme-mediated hydride transfer from reduced pyridine nucleotide cofactors to FAD [110]. NQO1 reduces coenzyme Q9 and coenzyme Q10 to their antioxidant hydroquinone forms and protects the cell from lipid peroxidation. Therefore, adipocytes have high levels of NQO1 expression. On the other hand, coenzyme Q10, which presents in the inner membrane of mitochondria, is an endogenous lipid-soluble antioxidant and has beneficial effects on diabetes-induced oxidative stress in rats. In addition, Coenzyme Q10 enhances Nrf2 mRNA levels and increases catalase activity [111]. Some proteins like p53, p63, p73, PGC1-α, and HIF-1α bind and are protected by NQO1 from proteasomal degradation, and NQO1 is protected against proteolytic digestion by NAD(P)H [109]. Nrf2 also mediates the expression of enzymes responsible for the refilling of the cytosolic pool of NADPH; glucose-6 phosphate dehydrogenase (G6PD), malic enzyme 1 (ME1), phosphogluconate dehydrogenase (PGD), and isocitrate dehydrogenase 1 (IDH1), NADPH is utilized for retaining and regeneration of cellular detoxifying [14].

### 3.3. Superoxide Dismutase

Superoxide Dismutase (SOD; EC 1.15.1.1) is an essential antioxidant enzyme for eliminating superoxide in cells’ cytosol, mitochondria, endoplasmic reticulum. In addition, SOD can detoxify nitrating agent peroxynitrite (ONOO^−^), produced by the reaction of superoxide anion with nitric oxide. SOD catalysis superoxide into H_2_O_2_ that is catalyzed by catalase or glutathione peroxidase enzymatic reactions [112]. It is a metalloenzyme with zinc (Zn), copper (Cu) and manganese (Mn) metal ions which are differently present in three SOD isoforms, however with a common mechanism of the action [113]. Cytoplasmic CuZnSOD (SOD1) is expressed as a prevalent isoform in all cells, mitochondrial MnSOD (SOD2) is considered the first line of defense against superoxide anion and extracellular CuZnSOD (SOD3) [114,115]. The human *sod1*, *sod2,* and *sod3* genes are localized on chromosome 21q22, chromosome 6q25.3, and chromosome 4, respectively. The *sod2* gene has a unique genetic organization and has little similarity with *sod1* and *sod3*. Various transcription factor-binding elements regulate human *sod* genes. The PI3K/Akt pathway can activate NF-κB and up-regulate SOD1 expression. NF-κB significantly up-regulates SOD2. In addition, Nrf2 protein can up-regulate *sod* genes through the antioxidant responsive element [116].

Retinal mitochondrial morphology alters during glucose-induced ROS production. However, some results have revealed that overexpression of SOD prevents the development of diabetic retinopathy by repealing mitochondrial dysfunction [117]. Under hyperglycemic condition, increasing the glucose flux promotes the production of superoxide anion O_2_**^.-^** in the mitochondrial electron-transport chain, which in turn induces the production of AGE products, activation of PKC and hexosamine pathways, and as a result the formation of secondary reactive oxygen species [118], including peroxynitrite and hydroxyl radicals [119]. Furthermore, SOD glycation occurs in diabetic conditions that lead to its structural and functional alteration and consequently decreased detoxification of superoxide anion potential and cell damage [120]. SOD can affect the autoxidation rates of hydroquinones produced by NQO1 either inhibit or accelerate, which is involved the reduction of a semiquinone by superoxide anion. SOD3 deficiency is observed in diabetic patients. One study indicates that the expression of SOD3 protein in the skin of diabetic patients is relatively low; hence, protection against oxidative stress is decreased [119].

### 3.4. Thioredoxin Reductase

The thioredoxin reductase system (TrxR; EC 1.8.1.9), consisting of three functional components; thioredoxin (Trx), TrxR, and NADPH, play a pivotal role in a wide range of cellular processes such as DNA synthesis, apoptosis, and cellular defense against ROS or RNS [121]. TrxR as a selenoenzyme contains three isoforms, TrxR1, TrxR2, and TrxR3 (TGR), which present primarily in the cytoplasm, mitochondria, and testis [122]. Trx is a small ubiquitous protein with two redox-active cysteine residues (-Cys-Gly-Pro-Cys-) at the active site. The most fundamental role of the Trx system is its function as a protein disulfide reductase. The cytoplasmic Trx/TrxR system maintains reduced protein and delivers electrons to some cellular reductases, including peroxiredoxins (Prdxs) [121]. They contain the peroxidatic cysteine residue that can react with H_2_O_2_, peroxynitrite, and lipid peroxides to produce a sulfenic acid form, which reacts with resolving cysteine and forms a disulfide bond. Afterward, thioredoxin reduces the disulfide bond, with the reducing equivalents NADPH provided by thioredoxin reductase (TrxR1) [123]. The copy numbers of electron carriers, such as thioredoxins (Trx), glutaredoxins (Grx), and GSH, increase in oxidative stress conditions along with antioxidant enzymes [124]. This system is involved in cellular redox signaling by modulating the activity of various transcription factors, such as NF-κB, p53, HIFα, PTEN, AP-1, FoxO, and Nrf2, thereby controlling various cellular processes [125]. Nrf2-Keap1, FoxO, and p53 transcription factors can detect oxidative stress and promote antioxidant gene expressions [126]. On the other hand, the Nrf2 system can be regulated by thioredoxin reductase 1(TrxR1) and the sirtuin family of deacetylases [127]. Moreover, Prdxs and glutathione peroxidases (GPx) remove H_2_O_2_ and help the regulation of its levels in the cell [128]. Inhibition of TrxR can be compensated by the up-regulation of Nrf2-dependent response, including increased HO-1 expression. Therefore, this reductase might be the critical selenoenzyme in regulating Nrf2 [129,130]. Furthermore, Nrf2 controls the expression of Trx by the regulation of TrxR1 and sulfiredoxin (Srxn1). TrxR1 and Srxn1 play are crucial for reducing oxidized protein thiols, including Trx and Prdxs [131]. Srxn1 catalyzes the ATP-dependent formation of a sulfinic acid phosphoric ester on Prdxs and is then reduced by Trx [106].

### 3.5. Glutathione Reductase

Glutathione reductase (GR; EC 1.8.1.7) is a homodimeric flavoprotein that controls cellular GSH homeostasis using NADPH as a reducing cofactor [132]. GR is a pivotal regulator for cell survival, and any dysfunction leads to various diseases [133]. Nrf2 regulates the synthesis and maintenance of the ubiquitous intracellular tripeptide thiol-containing glutathione. The reduced form of glutathione (GSH) is a predominant form of the molecule, and the disulfide-oxidized (GSSG) form consists of less than 1% of the cellular glutathione pool [134]. This tripeptide acts as an electron donor in reducing H_2_O_2_ to water and organic hydro-peroxides catalyzed by GPx [135]. Various glutathione biosynthetic enzymes, including glutathione cysteine ligase modifier subunit (GCLM) and glutathione cysteine ligase catalytic subunit (GCLC), GSH-dependent antioxidant enzymes (GPx2, glutathione S-transferases (GST), and heme oxygenase-1 (HO-1) are up-regulated in the Nrf2-driven expression of antioxidant enzymes [136]. Nrf2 has a fundamental role in maintaining cellular GSH balances through regulating various enzymes that use GSH as a cofactor [137], preserving intracellular GSH levels by regulating cysteine influx [138], and controlling the production of essential enzymes for GSH biosynthesis several mechanisms. Thereby, deficiency or lack of Nrf2 leads to a decrease in GSH levels [139,140]. Furthermore, it has been determined that the regulation of GSH redox status through the Nrf2 pathway occurs by GR-mediated recycling of oxidized glutathione. This mechanism takes precedence over the biosynthesis of enzymes involved in GSH synthesis [141].

### 3.6. Catalase

Catalase (CAT; EC 1.11.1.6) is a primary and robust defense component against ROS, decomposing hydrogen peroxide into water and oxygen [142]. Catalase has the highest turnover numbers among all enzymes. One molecule of catalase can break down even more than two million molecules of H_2_O_2_ per second in some sources [143]. Catalase is a tetrameric enzyme with one heme group and is dominantly present in the peroxisomes of mammalian cells. The liver, kidney, and erythrocytes show the highest catalase activity; contrary, serum, pancreas, and connective tissue show the lowest activity [144]. The mechanism involves oxidation of structural heme to an oxyferryl species by one molecule of H_2_O_2_; therefore, a porphyrin cation radical is produced. Then, the resting state of the enzyme occurs when a second hydrogen peroxide molecule acts as a reducing agent, and oxygen and water are produced [113]. The human catalase gene is located on the short arm of chromosome 13 and includes 13 exons and 12 introns [144]. Based on various studies, catalase malfunctioning or deficiency is associated with many diseases like diabetes mellitus. The genetic defects of erythrocyte catalase like acatalasemia and hypocatalasemia (less than 10% and 50% of the regular activity of catalase, respectively) may contribute to the development of diabetes [145,146]. H_2_O_2_ at low concentrations acts as a signaling molecule but is toxic on the cells at higher concentrations. Therefore, homeostasis maintaining of the cells by degradation of H_2_O_2_ is very vital. Furthermore, H_2_O_2_ acts as a secondary messenger in the signaling pathway for insulin release by inactivating tyrosine phosphatase. On the other hand, catalase eliminates ROS generation produced and promotes Nrf2 nuclear translocation and HO-1 gene expression in the mice model [147]. It has been described that the expression of catalase like SOD, GPx, NOQ-1, and HO-1 increases by Nrf2 activation. At the same time, Nrf2 deficiency significantly decreases the catalase expression [3,17,148]. Moreover, the elevated ROS would result in reduced catalase activity without affecting the expression of its mRNA [149]. Also, a reduction in Sirtuin1 and Nrf2 level is associated with a decrease in catalase and glutathione peroxidase [94]. According to the results obtained by Dinic et al., chemokine C-X-C Ligand 12 (CXCL12) can facilitate nuclear localization of Nrf2 and enhance its binding to the CAT gene promoter, prompting constitutive CAT expression and activity that was critical for defending β-cells from H_2_O_2_ toxicity [93]. More than fifteen transcription factors, including Nrf2 positively regulate the activity of the highly conserved core catalase promoter among species. At different binding site positions located at the distance range -45 to -11710 from ATG translation start codon [150]. Nowadays we know that Nrf2 is an important transcription factor regarding catalase expression. However, its precise involvement was controversial until five years ago, thitherto no ARE sequences had been reported in the catalase promoters, which calls into question Nrf2 binding directly to the catalase promoters and increasing catalase expression [150]. However, Dinic et al. provided conclusive evidence for the involvement of Nrf2 in the positive regulation of CAT gene transcription in rat pancreatic cells through the interaction between Nrf2 and the CAT gene promoter [93].

## 4. Keap1-Nrf2 Pathway and HIF-1 Activation in Diabetic Hypoxia-Induced Complications

**Figure 4:**
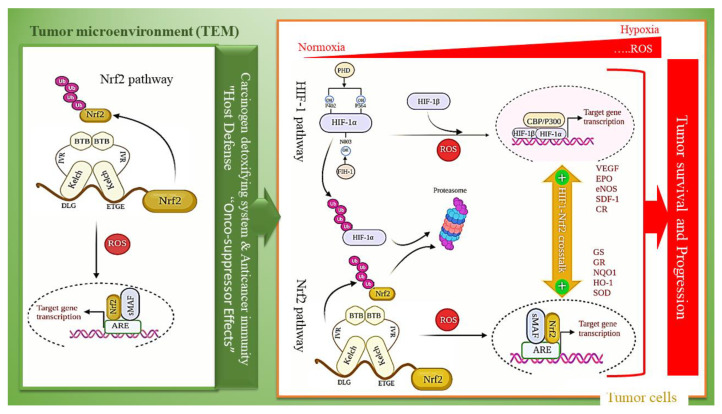
Dual-edge sward functioning of Nrf2 with tumor cells and crosstalk between Nrf2 and HIF-1 activation. Under hypoxia and in the presence of oxidative stress, Nrf2 and HIF1 both enhance the tumor malignancy in tumor cells, but Nrf2 activation in the tumor microenvironment (TME) triggers anticancer immunity, thus suppressing tumors.

The recent achievements in network medicine have significantly improved our awareness of multi-pathway interactions in complex diseases and may develop approaches to novel drug design. Oxygen (O_2_) is an essential factor in cell energy metabolism [151]. Eukaryotes generate adenosine triphosphate (ATP) link to the mitochondrial electron-transport chain (ETC) in which oxygen is the final electron acceptor. Due to its very high importance, the oxygen concentration must always be controlled in cells. HIF-1 as a transcription factor is a central regulator in response to hypoxia. Whereas HIF-1 is negatively regulated under normoxia and in the presence of O_2_. Nitric oxide (NO) nitrosylate HIF-1 to allow escaping HIF-1 from degradation under normoxia and HIF-1 increase NO production, including increasing the expression of the iNOS. HIF-1 is a heterodimer of a non-constitutively expressed HIF-1α subunit that its expression is regulated in response to the oxygen level. and constitutively expressed HIF-1β subunit, also known as aryl hydrocarbon receptor nuclear translocator (ARNT) [152]. At normoxic conditions, HIF-1α is rapidly modified then destructed. Its Modification occurs through proline and asparagine hydroxylation by prolyl hydroxylase domain proteins (PHDs) and factor inhibiting HIF-1 (FIH-1), respectively [153,154]. The poly-hydroxylated HIF-1 can be associated with the von Hippel-Lindau (VHL) protein, making it a candidate for polyubiquitination that leads to degradation in the proteasome. Under increased generation of ROS related to hypoxic cells (for example, within solid tumors), HIF-1α degradation is inhibited. Then HIF-1α is translocated to the nucleus to bind the hypoxia-responsive elements (HREs) on DNA in joint with HIF-1β as a heterodimer. The HIF-HRE binding increases transcription of hypoxia-responsive genes such as vascular endothelial growth factor (VEGF), erythropoietin (EPO), and various glycolytic enzymes [155]. Growing data also proposes that hypoxia’s cellular response is more intricate, containing consonant pathways through stress response pathways. The Nrf2 pathway is essential signaling that is also triggered in response to hypoxia and consequently ROS generation, involved in numerous cancer types [156], diabetes [157], and neurodegenerative diseases [158]. As summarized in Figure 4, there is mounting evidence that Nrf2 signaling plays a role in activating and sustaining the HIF-1 response. Evidences suggest crosstalk between Nrf2 and HIF-1 activation in which Nrf2 pathway plays a pivotal role in stimulating and satisfying the HIF-1-related response [159] and HIF-1α bolsters the Nrf2 signal. Some findings have revealed that Nrf2 or its downstream targets involved in the regulation of PHDs. Accordingly, knockdown of Nrf2 hinders HIF-1α accumulation in hypoxic cancer cells at post-translational levels, which subsequently suppresses the expression of hypoxia-inducible genes including metabolic enzymes [159,160,161]. Based on several studies, inhibition of Nrf2 leads to decreasing HIF-1α protein levels in colon cancer through stimulating proteasomal degradation and the suppression of glucose uptake in colon cancer cells [159]. NQO1 links to HIF-1α physically and reduce HIF-1α-PHDs interaction. Therefore, overexpression of NQO1 increases HIF-1α level and its half-life that indicates on an interactive and complex correlation between Nrf2 and the HIF-1 signaling pathways [162]. Nrf2 affect inflammation through direct and indirect mechanisms. In a direct mode, Nrf2 blocks the transcription of various cytokines, containing interleukin-6 (IL-6) and Interleukin-1β (IL-1β). While in indirect mechanism moderating ROS levels by improving the expression of antioxidant genes. High glucose levels lead to the destabilization of HIF-1α in hypoxia conditions through disruption the interaction between HIF-1α and p300, as a co-activator, and downregulate HIF-1 related responses [163]. As well, several studies have proved that ischemia has a close relationship to diabetic complications. Under hypoxia, production of endothelial nitric oxide synthase (eNOS), stromal cell-derived factor-1 (SDF-1), also known as chemokine CXCL12, chemokine receptors (CRs), vascular endothelial growth factor (VEGF), and other growth factors are increased, whereas, in the presence of high glucose in diabetic tissues, these factors are downregulated [52,164,165]. Therefore, HIF-1α induction and liberation of growth factors lead to reduce the tissue damages that occur in some ischemia-related diabetic complications as well as wound healing improvement in diabetic patients [166]. Both Nrf2 and HIF-1 pathways are notably related to chemoresistance in a different types of tumors. There is a metabolic change from the tricarboxylic acid cycle (TCA) to glycolysis in hypoxia conditions. This shift promotes chemoresistance via alterations in cellular metabolism.

ROS-dependent activation of Nrf2 promotes tumor progression in tumor cells, whereas tumor microenvironment (TME) has tumor-suppressor effects [167]. Accordingly, Nrf2 activation in tumor cells enhances their malignancy, but Nrf2 activation in TME of the tumor cells triggers anticancer immunity, thus suppressing tumors (Figure 3) [168]. The significance of the coordinated signaling and balance between Nrf2 activation under hypoxia-induced ROS generation in the tumor microenvironment (TME) and its activation in a synergistic mode with the Hif-1 pathway in tumor cells shade lights on the new views on cancer therapy to combat against tumor progression and resistance to therapy [169]. Nowadays, pharmacological attempts have focused on the oxidative modulation of tumors and the crosstalk between Nrf2 and Hif-1 pathways [167].

Moreover, oxidative stress shows a fundamental role in Alzheimer’s disease (AD) as a progressive degenerative disorder. Oxidative stress happens at the early stage of AD, and it prompts and activates several cell signaling pathways that are involved in neurodegeneration in AD [170,171]. Some studies have shown that oxidative stress induces Aβ production and aggregation, amplifying oxidative stress [172,173,174]. Therefore, oxidative stress-related Nrf2 activation or its activators can be therapeutically valuable in the treatment of neurodegenerative diseases, including AD, Parkinson’s disease (PD), and Amyotrophic lateral sclerosis [175].

## 5. Anti-Inflammatory Effects of Curcumin as a Multipotent Agent

Phytochemicals have potential effects on the modulation of intracellular processes. These compounds show significant therapeutic effects in various diseases through separate pathways. Curcumin is a yellow polyphenolic curcuminoid derived from *Curcuma longa* (turmeric) that has exposed several physiological and pharmacological properties, including antioxidant, anti-diabetic, anti-inflammatory, anti-carcinogenic, and neuroprotective properties. Also, curcumin is usually used in some countries as a nutritional spice [33]. Curcuminoids complex, which is characterized in turmeric, comprises curcumin, demethoxycurcumin [4-hydroxycinnamoyl-(4-hydroxy-3-methoxycinnamoyl) methane], and bisdemethoxycurcumin [bis-(4-hydroxy cinnamoyl) methane] [176]. Curcumin is a multi-target agent due to its unique structure. It contains enol and ketone groups that can react with the key cysteine thiolate residues in Keap1; thus, Cys151 of Keap1 is modified by curcumin that results in Keap1 conformational change to attenuate the Keap1–Nrf2 protein-protein interactions. Consequently, Nrf2 is translocated to the nucleus, interacts with the ARE region of the targets gene, then activates the transcriptional pathway to alleviate the cellular redox condition [33]. Numerous studies demonstrated that curcumin could modulate cellular targets, modify various cell signaling pathways, and downregulate the cell survival gene expression via transcription factors modulation [177]. Curcumin can regulate transcription factors like Nrf2, NF-ĸB, signal transducer and activator of transcription 3 (STAT3), protein kinases like the mammalian target of rapamycin, mitogen-activated protein kinases, and Akt. Therefore, curcumin affects PI3K/AKT/mTOR, Ras/Raf/MEK/ERK, GSK-3β pathways, activates the p53 pathway, and regulates survival pathways via NF-κB, Akt, and Nrf2/ARE pathways [33,178]. In addition, curcumin could constraint the production of pro-inflammatory monocyte/macrophage-derived cytokines including interleukin-1 (IL-1), interleukin-6 (IL-6), interleukin-8 (IL-8), interleukin-1b (IL-1b), and tumor necrosis factor-α (TNF-α). Curcumin could also suppress lipo-oxygenase-5 (LOX5) and cyclo-oxygenase-2 (COX2), prostaglandin E2 (PGE2), heme oxygenase-1 (HO-1), xanthine oxygenase activities, aspartate aminotransferase, alanine aminotransferase, nitric oxide synthesis, and could act against ROS generation [179,180,181,182]. Therefore, curcumin can suppress acute and chronic inflammation, reduce the inflammatory response, and improve energy homeostasis through direct and indirect antioxidant effects by eliminating ROS and RNS. Hence, curcumin via a wide variety of valuable activities could be used as a therapeutic agent in various diseases like diabetes [183].

## 6. Targeting of Keap1-Nrf2 and Related Pathways by Curcumin in Diabetes

**Figure 5:**
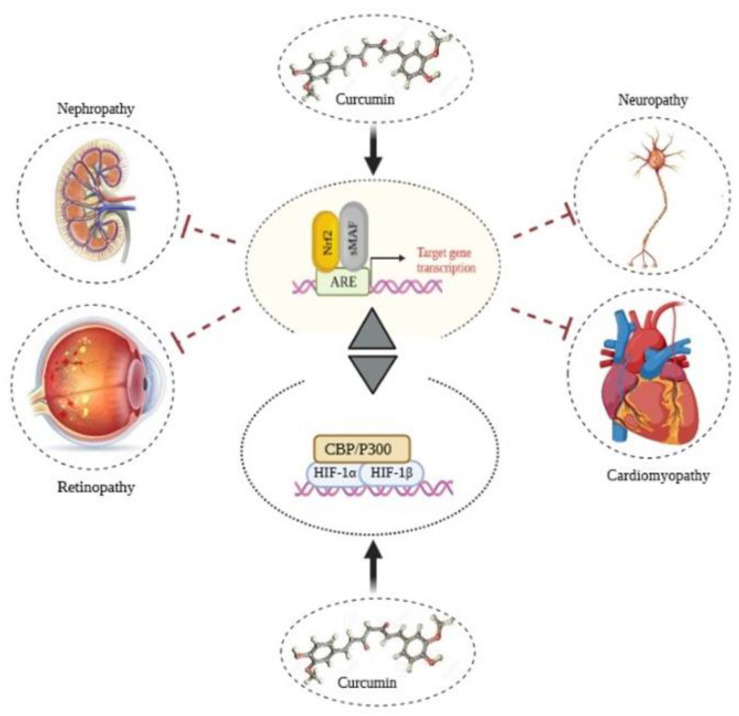
Anti-inflammatory effects of curcumin through modulation on Nrf2 and HIF1. Considering their correlation, modulation of Nrf2 and HIF-1 signaling pathways reduces diabetic complications by balancing ROS levels.

Diabetes has now converted an epidemic of the 21st century, and it is estimated that 642 million people will be infected by 2040 [184]. This multilateral disease leads to approximately 5 million deaths every year (one death every 6 s). Due to impaired production and function of insulin, the circulating glucose level is increased, resulting in hyperglycemia and diabetic microvascular and macrovascular complications. The microvascular complications include damage to blood vessels providing neurons, nephrons, and retina. Hyperglycemia-induced oxidative stress and related pathways have a fundamental role in the pathogenesis of diabetic vascular complications. Therefore, extended hyperglycemia is one of the main reasons for various diabetic complications, including cardiomyopathy, retinopathy, nephropathy, and neuropathy. Under hyperglycemic conditions, AGEs are generated, which affect multiple target cells via various receptors, leading to oxidative stress induction [185,186,187,188]. Nrf2-related response to oxidative stress can decrease oxidative stress by modulating antioxidant enzyme activity in cells. Regulation of the Keap1-Nrf2 pathway occurs in different steps via various signaling cascades and factors. Several studies have shown curcumin act as a pivotal regulator for Nrf2 and related pathways; it, as a multi-target molecule, has revealed extensive performance directly and indirectly in targeting different biological processes (Figure 5). According to the large body of findings, curcumin has been used to treat diabetes and moderate its complications through targeting several pathways, which are discussed separately in the following sections.

### 6.1. Diabetic Cardiomyopathy

Diabetic cardiomyopathy (DCM) is one of the most critical cardiac complications categorized by structural and functional disorders in the myocardium of diabetic patients [189,190]. Hyperglycemia and AGEs production in diabetic patients leads to endothelial dysfunction, fibrosis, amplified inflammation, and oxidative stress (5%). Various reports from extensive applications have shown that curcumin can deal with hyperglycemia, insulin resistance, obesity, and related metabolic disorders. Antioxidant and anti-inflammatory activity of curcumin is associated with ROS removal in cells and suppression of NF-κB signaling pathway, respectively [191]. A large body of findings shows that DCM pathogenesis correlates with several factors and pathways, including PKC signaling pathway, activation of JNKs, a prominent member of MAPK, and activation of advanced AGEs. Curcumin modulate with numerous targets such as PKC, JNK, Nrf2, and peroxisome proliferator-activated receptor-γγ(PPARγ). Curcumin can suppress the production of TNF-α and related cell signaling pathways such as NF-κB signaling [192,193]. Also, curcumin could reduce overexpressed inflammatory factors and inhibit the toll-like receptor (TLR)4-MAPK/NFκB signaling pathway [194]. Due to the structure of curcumin, it could act as a natural ROS scavenger. Curcumin could effectively increase Nrf2-related expression of antioxidant enzymes including catalase, superoxide dismutase, and glutathione-S-transferase in diabetic rats [193]. Furthermore, it considerably stopped diabetes-induced translocation of PKCα and PKCβ2 to membranous section and efficiently inhibited p38 MAPK and ERK1/2 pathways in the left ventricular tissue of diabetic rats [195]. Also, insulin release, HO-1 gene expression and its activity were considerably improved when Langerhans cells were treated with curcumin [196]. Curcumin and its derivatives considerably upregulated HO-1 expression and activity in the cardiac tissue of diabetic rats [197]. In vitro treatment of curcumin could effectively suppress cardiomyocyte apoptosis through oxidative stress-reducing maintenance of Akt and GSK3β phosphorylation [198]. As well, the Nrf2 pathway shows a fundamental role in protecting diabetes-induced aortic injury [199].

### 6.2. Diabetic Retinopathy

Diabetic patients show various ophthalmic complications; counting retinopathy, glaucoma, and cataracts which Diabetic retinopathy (DR) is the most severe and destructive among them [200]. The global prevalence of blindness is estimated to be 1.5 billion, of which 0.4 million is due to DR. It has been estimated that worldwide, about 93 million diabetic patients have DR among them 17 million with proliferative DR, 21 million with diabetic macular edema, 28 million with vision-threatening diabetic retinopathy (VTDR). Thereby this complication affects 1 in 3 persons with diabetes and remains the leading cause of blindness [201]. High glucose levels in diabetic patients affect the retina and its microvasculature slowly and eventually destroy them. In addition to hyperglycemia, other leading causes containing hypertension and hyperlipidemia also exacerbate diabetic retinopathy. Hyperglycemia leads to a disparity between production and demolition of ROS, and metabolic changes raise ROS production in retina cells. ROS causes activation of PKC, polyol, and hexosamine pathways and accumulation of AGEs [202,203]. All of these pathways are participate in the pathogenesis of diabetic retinopathy. Stimulation of the polyol pathway alters glucose into polyols, which uses NADPH. Thereupon, decreasing the NADPH levels reduces its accessibility for the biosynthesis of intracellular antioxidant glutathione that it is so destructive to the antioxidant defense system [204]. Also, activating PKC by a high diacyl glycerol level via activating NADPH oxidase can promote ROS production in cells [205]. Furthermore, glycation of macromolecules and AGEs generation can activate NADPH oxidase, ROS, and oxidative stress. As mentioned above, the Nrf2 related antioxidant system is the fundamental pathway for dealing with oxidative stress. In diabetes, Nrf2 expression and its binding with Keap1 are augmented in the retina as well as DNA binding activity of Nrf2 and GSH levels are reduced [206,207,208]. Therefore, the Keap1-Nrf2 pathway targeting diabetic retinopathy is a precise molecular approach to this complication.

A study shows that curcumin raises Nrf2 expression by demethylating its promoter [209]. Numerous studies determined curcumin’s effects on Nrf2 and related pathways as an inducer. This multi-target molecule has been identified to be an efficient scavenger of ROS and utilizes cytoprotective properties in cultured retinal pigmented epithelial (RPE) cells [210,211,212]. Also, it has been clarified that curcumin has a pivotal role in activating the HO-1 gene and regulating the Nrf2 pathway and is related to the improvement of cell survival [213,214]. Also, some studies have specified that curcumin can effectively reduce retinal vascular leakage in an animal model of DR [93]. A large body of studies has shown that activation of Nrf2 pathway can be increase through the maintenance and/or phosphorylation of the Nrf2 protein and correlated to various signaling proteins, containing the MAPKs signaling pathway [42,44]. Also, based on Bucolo et al. study, curcumin mediates ERK1/2 phosphorylation and upregulation of Nrf2 and related gene such as HO-1 to promote survival of RPE cells [215].

### 6.3. Diabetic Nephropathy

Diabetic nephropathy (DNeph) is the essential basis of progressive kidney disease and shows high incidence worldwide. DNeph occurs in 20–40% of all diabetic patients, and it is the primary cause of end-stage renal disease (ESRD) [216,217]. DNeph still can not be efficiently treated, so it is crucial to improve more effective medicines to prevent or slow its development [218]. Oxidative stress via the inflammatory process can promote impairment of renal function. Therefore, inhibiting hyperglycemia-induced oxidative stress and inflammatory responses are attractive strategies for treating DNeph and increasing impressive therapeutic drugs. Nrf2 related gene activation, as a pivotal pathway to reducing oxidative stress and consequences, is an effective mechanism to reducing diabetic nephropathy. Curcumin as a multi target molecule can reduce kidney damage in diabetic rats through stimulating the Nrf2 pathway, preventing Nf-κB signaling, suppressing NADPH oxidase, and downregulating PKC [175]. Based on Bo Hwan Kim et al. study, curcumin improved albuminuria, pathophysiologic disorders on the glomerulus, urinary malondialdehyde (MDA), and urinary SOD related with induced Nrf2 pathway. Also, it exerts therapeutic effects on lipid accumulation and prompts oxidative stress associated with AMPK activation and its downstream factors in DNeph in rats [219]. It has been reported that the activation of the Nrf2 pathway reduces DNeph progression by reducing oxidative stress and inflammatory mediators [220]. Curcumin has been verified to be a potent Nrf2 activator in animal studies [221].

Since curcumin activates Nrf2-related antioxidant enzymes to reveal cytoprotective properties [222,223], its usage is considered an effective DNeph prevention strategy. Based on Balogun et al. study, curcumin disturbed the Nrf2–Keap1 complex and increased the expression and activity of HO-1in porcine renal epithelial proximal tubule cells. Also, curcumin treatment has been revealed to reduce macrophage infiltration in the kidneys of chronic renal disorder rats and suppression of NF-κB signaling [224]. Another study showed that using curcumin for 16 weeks in diabetic mice decreased renal hypertrophy, extracellular matrix extension, and albuminuria levels [225]. Curcumin can suppress PKC activity and attenuate TGF-β1 as a connective growth factor and extracellular matrix proteins, including fibronectin and collagen IV [221].

### 6.4. Diabetic Neuropathy

Diabetic neuropathy (DNeu) is an inflammation-related neurodegenerative disease (ND) affecting approximately 60% of diabetics worldwide [226]. An essential transcription factor involved in regulating the expression of various inflammatory proteins is NF-κB as an important player in DNeu pathogenesis [227]. Moreover, a large body of finding revealed that neuronal dysfunction correlates thoroughly with the activation of NF-κB and the expression of pro-inflammatory cytokines, including IL-6 and TNF-α [228,229,230]. Increased AGEs in hyperglycemia can promote upregulation of NF-κB and its related gene expression in the peripheral nerves of diabetic mice [231]. Curcumin, via anti-inflammatory effect, reduces inflammation and consequences in various cells. Also, based on multiple studies, curcumin can reduce thermal hyperalgesia in a mouse model of diabetic neuropathic pain mediated via inhibition of TNF-α and NO release [232,233]. Curcumin derivatives reduce TNFα activation and other neuro-inflammation factors in the CNS through activation of the AMPK signaling pathway [234]. Zhao et al.’s study show that curcumin attenuates neuropathic pain in diabetic rats by inhibiting NADPH-oxidase-mediating oxidative stress in the spinal cord [235]. Curcumin up-regulates the expression of SOD (GPx) and CAT [97]; as a result, it decreases lipid peroxidation and MDA production in treated cells [236]. On the other hand, it can down-regulate NF-κB and level of inflammatory factors like TNF-α, IL-6, and interleukin-1β (IL-1β) [237].

## 7. Future Perspective and Conclusions

It is appropriate to conclude that the Keap1-Nrf2 pathway has a fundamental role in the onset and progress of various inflammatory diseases. Modulation of Keap1-Nrf2 directly or indirectly can be a potential strategy in establishing normal cellular conditions. The pivotal role of this pathway and its correlation with various signaling pathways and factors indicates an extensive internal network in the development of different diseases; according to the description provided in this review, some diseases and their complications are directly related to oxidative stress in cells. Diabetic complications associated with oxidative stress can be significantly reduced by curcumin, which targets numerous pathways in the cells as a therapeutic strategy. The application of multifunctional agents can effectively increase the ability of cells to deal with metabolic disorders. A large body of evidence highlights the role of curcumin in reducing diseases and their complications. Curcumin as a multi-target agent has the ability to modulate various cellular signaling pathways in order to create stable cellular conditions. Furthermore, this ability is much more evident in relation to functional synergies with other natural or synthetic compounds. Further studies on synergistic approaches to curcumin activity in the presence of other compounds and the study of its possible mechanisms could be one of the most interesting topics for future studies.

## Figures and Tables

**Table 1:** Keap1/Nrf2/ARE relevant downstream enzymes.

Enzyme	EC Number *	Substrates	Refs.
Heme oxygenase-1 (HO-1)	1.14.14.18	Heme, NAD(P)H, O_2_	[85,86]
NADPH-quinone oxidoreductase-1 (NQO-1)	1.6. 5.2	Quinone, NAD(P)H	[85]
Superoxide dismutase (SOD)	1.15.1.1	O_2_^◦−^	[87]
Thioredoxin reductase (TrxR)	1.8.1.9	Thioredoxin, NAD(P)H	[88,89,90]
Glutathione reductase (GR)	1.8.1.7	GSSG, NAD(P)H	[91]
Catalase (CAT)	1.11.1.6	H_2_O_2_	[92,93]
Glutathione peroxidase (GPx)	1.11.1.9	H_2_O_2_, GSH	[94]
Glutamate-cysteine ligase (GCL)	6.3.2.2	L.glutamate, L-cysteine, ATP	[95]
Glutathione synthase (GSS)	6.3.2.3	Gamma-l-glutamyl-l-cysteine, Glycine	[96,97]
Glutathione S-transferase (GST)	2.5.1.18	GSH, Xenobiotic substrates	[98]
Peroxiredoxins (PRDX1)	1.11.1.15	H_2_O_2_, Organic hydroperoxides, peroxynitrite	[99]
UDP-glucuronosyl transferase (UGT)	2.4.1.17	Aliphatic alcohols, phenols, carboxylic acids, thiols, and amines	[100,101]
Sulfiredoxin1 (SRXN1)	1.8.98.2	Peroxiredoxin, ATP, and thiols	[102]

* BRENDA, the ELIXIR core data resource in 2021: new developments and updates [101].

## Abbreviations

AD	Alzheimer’ s disease
AGC	cAMP-dependent, cGMP-dependent and protein kinase C
AGEs	advanced glycation end-products
AMPK	AMP-activated protein kinase
ARE	antioxidant response element
BCR	BTB-CUL3-RBX1
BTB	bric-a-brac, tram-track and broad-complex
CAT	Catalase
CBP	CREB-binding protein
CDK5	cyclin-dependent kinase 5
CREB	cAMP-response element-binding protein
ERK	extracellular signal-regulated kinases
GCLC	glutathione cysteine ligase modifier subunit
GPx	glutathione peroxidase
GR	glutathione reductase
GSH	Glutathione
GSK3	glycogen synthase kinase 3
HIF-1	hypoxia-inducible factor 1
HO-1	heme oxygenase-1
IDF	International Diabetes Federation
iNOS	inducible nitric oxide synthase
IRS1	receptor substrate-1
JNK	cJun N-terminal kinases
Keap1	kelch-like-ECH-associated protein 1
MAPKs	mitogen-activated protein kinases
Neh	Nrf2-ECH homology
NF-κB	nuclear factor kappa-light-chain-enhancer of activated B
NQO-1	NAD(P)H- quinone oxidoreductase-1
Nrf2	nuclear factor erythroid 2-related factor 2
PERK	protein kinase RNA-like ER kinase
PI3K	phosphatidylinositol-3 phosphate
PI3K	phosphoinositide 3-kinases
PIP3	phosphatidyl inositol triphosphate
PKC	protein kinase C
RNS	reactive nitrogen species
ROS	reactive oxygen species
GSH	glutathione
RXRα	retinoid x receptor α
sMAF	small musculoaponeurotic fibrosarcoma
SOD	superoxide dismutase
SPAK	stress-activated protein kinases
T1DM	type 1 diabetes
T2DM	type 2 diabetes
TEM	tumor microenvironment
Trx	thioredoxin
TrxR	thioredoxin reductase
β-TrCP	β-transducin repeat-containing protein

## References

[1] Di Meo S., Reed T.T., Venditti P., Victor V.M. (2016). Role of ROS and RNS Sources in Physiological and Pathological Conditions. Oxid. Med. Cell. Longev..

[2] Phaniendra A., Jestadi D.B., Periyasamy L. (2015). Free radicals: Properties, sources, targets, and their implication in various diseases. Indian J. Clin. Biochem..

[3] Jung K.-A., Kwak M.-K. (2010). The Nrf2 System as a Potential Target for the Development of Indirect Antioxidants. Molecules.

[4] Saha S., Buttari B., Panieri E., Profumo E., Saso L. (2020). An overview of Nrf2 signaling pathway and its role in inflammation. Molecules.

[5] Himmelfarb J., Hakim R.M. (2003). Oxidative stress in uremia. Curr. Opin. Nephrol. Hypertens..

[6] Himmelfarb J., Stenvinkel P., Ikizler T.A., Hakim R.M. (2002). The elephant in uremia: Oxidant stress as a unifying concept of cardiovascular disease in uremia. Kidney Int..

[7] Moradi H., Pahl M.V., Elahimehr R., Vaziri N.D. (2009). Impaired antioxidant activity of high-density lipoprotein in chronic kidney disease. Transl. Res..

[8] Vaziri N.D. (2004). Oxidative stress in uremia: Nature, mechanisms, and potential consequences. Semin. Nephrol..

[9] Vaziri N.D., Moradi H., Pahl M.V., Fogelman A.M., Navab M. (2009). In vitro stimulation of HDL anti-inflammatory activity and inhibition of LDL pro-inflammatory activity in the plasma of patients with end-stage renal disease by an apoA-1 mimetic peptide. Kidney Int..

[10] Sies H., Jones D.P. (2020). Reactive oxygen species (ROS) as pleiotropic physiological signalling agents. Nat. Rev. Mol. Cell Biol..

[11] Sharifi-Rad M., Anil Kumar N.V., Zucca P., Varoni E.M., Dini L., Panzarini E., Rajkovic J., Tsouh Fokou P.V., Azzini E., Peluso I. (2020). Lifestyle, Oxidative Stress, and Antioxidants: Back and Forth in the Pathophysiology of Chronic Diseases. Front. Physiol..

[12] Ramachandran A., Das A., Joshi S., Yajnik C., Shah S., Kumar K.P. (2010). Current status of diabetes in India and need for novel therapeutic agents. J. Assoc. Physicians India.

[13] Atlas D. (2015). International diabetes federation. IDF Diabetes Atlas.

[14] Baumel-Alterzon S., Katz L.S., Brill G., Garcia-Ocaña A., Scott D.K. (2021). Nrf2: The Master and Captain of Beta Cell Fate. Trends Endocrinol. Metab..

[15] Nooshi-Nedamani S., Habibi-Rezaei M., Farzadfard A., Moosavi-Movahedi A. (2019). Intensification of serum albumin amyloidogenesis by a glycation-peroxidation loop (GPL). Arch. Biochem. Biophys..

[16] Huang Y., Li W., Su Z.-Y., Kong A.-N.T. (2015). The complexity of the Nrf2 pathway: Beyond the antioxidant response. J. Nutr. Biochem..

[17] Kopacz A., Kloska D., Forman H.J., Jozkowicz A., Grochot-Przeczek A. (2020). Beyond repression of Nrf2: An update on Keap1. Free Radic. Biol. Med..

[18] Kobayashi M., Yamamoto M. (2005). Molecular mechanisms activating the Nrf2-Keap1 pathway of antioxidant gene regulation. Antioxid. Redox Signal..

[19] Li R., Jia Z., Zhu H. (2019). Regulation of Nrf2 Signaling. React. Oxyg Species.

[20] Kobayashi M., Yamamoto M. (2006). Nrf2-Keap1 regulation of cellular defense mechanisms against electrophiles and reactive oxygen species. Adv. Enzym. Regul..

[21] Surh Y.-J., Kundu J.K., Na H.-K. (2008). Nrf2 as a master redox switch in turning on the cellular signaling involved in the induction of cytoprotective genes by some chemopreventive phytochemicals. Planta Med..

[22] Gupta S.C., Prasad S., Kim J.H., Patchva S., Webb L.J., Priyadarsini I.K., Aggarwal B.B. (2011). Multitargeting by curcumin as revealed by molecular interaction studies. Nat. Prod. Rep..

[23] Calabrese V., Bates T.E., Mancuso C., Cornelius C., Ventimiglia B., Cambria M.T., Di Renzo L., De Lorenzo A., Dinkova-Kostova A.T. (2008). Curcumin and the cellular stress response in free radical-related diseases. Mol. Nutr. Food Res..

[24] Kannappan R., Gupta S.C., Kim J.H., Reuter S., Aggarwal B.B. (2011). Neuroprotection by spice-derived nutraceuticals: You are what you eat!. Mol. Neurobiol..

[25] Esmaili M., Ghaffari S.M., Moosavi-Movahedi Z., Atri M.S., Sharifizadeh A., Farhadi M., Yousefi R., Chobert J.-M., Haertlé T., Moosavi-Movahedi A.A. (2011). Beta casein-micelle as a nano vehicle for solubility enhancement of curcumin; food industry application. Lwt-Food Sci. Technol..

[26] Mofidi-Najjar F., Taghavi F., Ghadari R., Sheibani N., Moosavi-Movahedi A.A. (2017). Destructive effect of non-enzymatic glycation on catalase and remediation via curcumin. Arch. Biochem. Biophys..

[27] Panieri E., Telkoparan-Akillilar P., Suzen S., Saso L. (2020). The NRF2/KEAP1 axis in the regulation of tumor metabolism: Mechanisms and therapeutic perspectives. Biomolecules.

[28] Itoh K., Chiba T., Takahashi S., Ishii T., Igarashi K., Katoh Y., Oyake T., Hayashi N., Satoh K., Hatayama I. (1997). An Nrf2/small Maf heterodimer mediates the induction of phase II detoxifying enzyme genes through antioxidant response elements. Biochem. Biophys. Res. Commun..

[29] Li W., Kong A.N. (2009). Molecular mechanisms of Nrf2-mediated antioxidant response. Mol. Carcinog..

[30] Wang H., Liu K., Geng M., Gao P., Wu X., Hai Y., Li Y., Li Y., Luo L., Hayes J.D. (2013). RXRα inhibits the NRF2-ARE signaling pathway through a direct interaction with the Neh7 domain of NRF2. Cancer Res..

[31] Luo Y., Eggler A.L., Liu D., Liu G., Mesecar A.D., van Breemen R.B. (2007). Sites of alkylation of human Keap1 by natural chemoprevention agents. J. Am. Soc. Mass Spectrom..

[32] Robledinos-Antón N., Fernández-Ginés R., Manda G., Cuadrado A. (2019). Activators and Inhibitors of NRF2: A Review of Their Potential for Clinical Development. Oxid. Med. Cell. Longev..

[33] Rahban M., Habibi-Rezaei M., Mazaheri M., Saso L., Moosavi-Movahedi A.A. (2020). Anti-Viral Potential and Modulation of Nrf2 by Curcumin: Pharmacological Implications. Antioxidants.

[34] Emanuele S., Celesia A., D’Anneo A., Lauricella M., Carlisi D., De Blasio A., Giuliano M. (2021). The Good and Bad of Nrf2: An Update in Cancer and New Perspectives in COVID-19. Int. J. Mol. Sci..

[35] Manning G., Whyte D.B., Martinez R., Hunter T., Sudarsanam S. (2002). The protein kinase complement of the human genome. Science.

[36] Liu Z., Lv Y., Zhao N., Guan G., Wang J. (2015). Protein kinase R-like ER kinase and its role in endoplasmic reticulum stress-decided cell fate. Cell Death Dis..

[37] Kaufman R.J., Scheuner D., Schröder M., Shen X., Lee K., Liu C.Y., Arnold S.M. (2002). The unfolded protein response in nutrient sensing and differentiation. Nat. Rev. Mol. Cell Biol..

[38] Liu Z.-W., Zhu H.-T., Chen K.-L., Dong X., Wei J., Qiu C., Xue J.-H. (2013). Protein kinase RNA-like endoplasmic reticulum kinase (PERK) signaling pathway plays a major role in reactive oxygen species (ROS)-mediated endoplasmic reticulum stress-induced apoptosis in diabetic cardiomyopathy. Cardiovasc. Diabetol..

[39] Rosse C., Linch M., Kermorgant S., Cameron A.J., Boeckeler K., Parker P.J. (2010). PKC and the control of localized signal dynamics. Nat. Rev. Mol. Cell Biol..

[40] Bloom D.A., Jaiswal A.K. (2003). Phosphorylation of Nrf2 at Ser40 by protein kinase C in response to antioxidants leads to the release of Nrf2 from INrf2, but is not required for Nrf2 stabilization/accumulation in the nucleus and transcriptional activation of antioxidant response element-mediated NAD (P) H: Quinone oxidoreductase-1 gene expression. J. Biol. Chem..

[41] Litchfield D.W. (2003). Protein kinase CK2: Structure, regulation and role in cellular decisions of life and death. Biochem. J..

[42] Apopa P.L., He X., Ma Q. (2008). Phosphorylation of Nrf2 in the transcription activation domain by casein kinase 2 (CK2) is critical for the nuclear translocation and transcription activation function of Nrf2 in IMR-32 neuroblastoma cells. J. Biochem. Mol. Toxicol..

[43] Ke Q., Yang J., Liu H., Huang Z., Bu L., Jin D., Liu C. (2021). Dose-and time-effects responses of Nonylphenol on oxidative stress in rat through the Keap1-Nrf2 signaling pathway. Ecotoxicol. Environ. Saf..

[44] Pi J., Bai Y., Reece J.M., Williams J., Liu D., Freeman M.L., Fahl W.E., Shugar D., Liu J., Qu W. (2007). Molecular mechanism of human Nrf2 activation and degradation: Role of sequential phosphorylation by protein kinase CK2. Free Radica. Biol. Med..

[45] Thomas S.M., Brugge J.S. (1997). Cellular functions regulated by Src family kinases. Annu. Rev. Cell Dev. Biol..

[46] Jain A.K., Jaiswal A.K. (2006). Phosphorylation of tyrosine 568 controls nuclear export of Nrf2. J. Biol. Chem..

[47] Fão L., Mota S.I., Rego A.C. (2019). c-Src regulates Nrf2 activity through PKCδ after oxidant stimulus. Biochim. Biophys. Acta Mol. Cell Res..

[48] Niture S.K., Jain A.K., Shelton P.M., Jaiswal A.K. (2011). Src subfamily kinases regulate nuclear export and degradation of transcription factor Nrf2 to switch off Nrf2-mediated antioxidant activation of cytoprotective gene expression. J. Biol. Chem..

[49] Jain A.K., Jaiswal A.K. (2007). GSK-3β acts upstream of Fyn kinase in regulation of nuclear export and degradation of NF-E2 related factor 2. J. Biol. Chem..

[50] Tsui H., Zeng Q., Chen K., Zhang X., Chackalamannil S., Rotella D., Ward S.E. (2017). 7.10-Inhibiting Kinases in the CNS. Comprehensive Medicinal Chemistry III.

[51] Jimenez-Blasco D., Santofimia-Castaño P., Gonzalez A., Almeida A., Bolaños J.P. (2015). Astrocyte NMDA receptors’ activity sustains neuronal survival through a Cdk5–Nrf2 pathway. Cell Death Differ..

[52] Gallagher K.A., Liu Z.-J., Xiao M., Chen H., Goldstein L.J., Buerk D.G., Nedeau A., Thom S.R., Velazquez O.C. (2007). Diabetic impairments in NO-mediated endothelial progenitor cell mobilization and homing are reversed by hyperoxia and SDF-1α. J. Clin. Investig..

[53] Sabio G., Davis R.J. (2014). Seminars in immunology. TNF and MAP Kinase Signalling Pathways.

[54] Paul A., Wilson S., Belham C.M., Robinson C.J., Scott P.H., Gould G.W., Plevin R. (1997). Stress-activated protein kinases: Activation, regulation and function. Cell. Signal..

[55] Brancho D., Tanaka N., Jaeschke A., Ventura J.-J., Kelkar N., Tanaka Y., Kyuuma M., Takeshita T., Flavell R.A., Davis R.J. (2003). Mechanism of p38 MAP kinase activation in vivo. Genes Dev..

[56] Enslen H., Brancho D.M., Davis R.J. (2000). Molecular determinants that mediate selective activation of p38 MAP kinase isoforms. Embo J..

[57] Remy G., Risco A.M., Iñesta-Vaquera F.A., González-Terán B., Sabio G., Davis R.J., Cuenda A. (2010). Differential activation of p38MAPK isoforms by MKK6 and MKK3. Cell. Signal..

[58] Kumar A., Mittal R. (2017). Nrf2: A potential therapeutic target for diabetic neuropathy. Inflammopharmacology.

[59] Paladino S., Conte A., Caggiano R., Pierantoni G.M., Faraonio R. (2018). Nrf2 Pathway in Age-Related Neurological Disorders: Insights into MicroRNAs. Cell. Physiol. Biochem. Int. J. Exp. Cell. Physiol. Biochem. Pharmacol..

[60] Yuan X., Xu C., Pan Z., Keum Y.S., Kim J.H., Shen G., Yu S., Oo K.T., Ma J., Kong A.N.T. (2006). Butylated hydroxyanisole regulates ARE-mediated gene expression via Nrf2 coupled with ERK and JNK signaling pathway in HepG2 cells. Mol. Carcinog..

[61] Kaidanovich-Beilin O., Woodgett J.R. (2011). GSK-3: Functional insights from cell biology and animal models. Front. Mol. Neurosci..

[62] Linding R., Jensen L.J., Ostheimer G.J., van Vugt M.A., Jørgensen C., Miron I.M., Diella F., Colwill K., Taylor L., Elder K. (2007). Systematic discovery of in vivo phosphorylation networks. Cell.

[63] Ghareghomi S., Ahmadian S., Zarghami N., Kahroba H. (2021). Fundamental insights into the interaction between telomerase/TERT and intracellular signaling pathways. Biochimie.

[64] Sun X., Chen L., He Z. (2019). PI3K/Akt-Nrf2 and anti-inflammation effect of macrolides in chronic obstructive pulmonary disease. Curr. Drug Metab..

[65] Ali T., Kim T., Rehman S.U., Khan M.S., Amin F.U., Khan M., Ikram M., Kim M.O. (2018). Natural dietary supplementation of anthocyanins via PI3K/Akt/Nrf2/HO-1 pathways mitigate oxidative stress, neurodegeneration, and memory impairment in a mouse model of Alzheimer’s disease. Mol. Neurobiol..

[66] Salazar M., Rojo A.I., Velasco D., de Sagarra R.M., Cuadrado A. (2006). Glycogen synthase kinase-3β inhibits the xenobiotic and antioxidant cell response by direct phosphorylation and nuclear exclusion of the transcription factor Nrf2. J. Biol. Chem..

[67] Chowdhry S., Zhang Y., McMahon M., Sutherland C., Cuadrado A., Hayes J.D. (2013). Nrf2 is controlled by two distinct β-TrCP recognition motifs in its Neh6 domain, one of which can be modulated by GSK-3 activity. Oncogene.

[68] McMahon M., Thomas N., Itoh K., Yamamoto M., Hayes J.D. (2004). Redox-regulated turnover of Nrf2 is determined by at least two separate protein domains, the redox-sensitive Neh2 degron and the redox-insensitive Neh6 degron. J. Biol. Chem..

[69] Rada P., Rojo A.I., Evrard-Todeschi N., Innamorato N.G., Cotte A., Jaworski T., Tobón-Velasco J.C., Devijver H., García-Mayoral M.F., Van Leuven F. (2012). Structural and functional characterization of Nrf2 degradation by the glycogen synthase kinase 3/β-TrCP axis. Mol. Cell. Biol..

[70] Unoki T., Akiyama M., Kumagai Y., Gonçalves F.M., Farina M., da Rocha J.B.T., Aschner M. (2018). Molecular Pathways Associated With Methylmercury-Induced Nrf2 Modulation. Fron. Genet..

[71] Bryan H.K., Olayanju A., Goldring C.E., Park B.K. (2013). The Nrf2 cell defence pathway: Keap1-dependent and-independent mechanisms of regulation. Biochem. Pharmacol..

[72] Sun Z., Chin Y.E., Zhang D.D. (2009). Acetylation of Nrf2 by p300/CBP augments promoter-specific DNA binding of Nrf2 during the antioxidant response. Mol. Cell. Biol..

[73] Birbach A., Gold P., Binder B.R., Hofer E., de Martin R., Schmid J.A. (2002). Signaling molecules of the NF-κB pathway shuttle constitutively between cytoplasm and nucleus. J. Biol. Chem..

[74] Yerra V.G., Negi G., Sharma S.S., Kumar A. (2013). Potential therapeutic effects of the simultaneous targeting of the Nrf2 and NF-κB pathways in diabetic neuropathy. Redox Biol..

[75] Lee S., Choi S.-Y., Choo Y.-Y., Kim O., Tran P.T., Dao C.T., Min B.-S., Lee J.-H. (2015). Sappanone A exhibits anti-inflammatory effects via modulation of Nrf2 and NF-κB. Int. Immunopharmacol..

[76] Liu G.-H., Qu J., Shen X. (2008). NF-κB/p65 antagonizes Nrf2-ARE pathway by depriving CBP from Nrf2 and facilitating recruitment of HDAC3 to MafK. Biochim. Biophys. Acta (Bba)-Mol. Cell Res..

[77] Eades G., Yang M., Yao Y., Zhang Y., Zhou Q. (2011). miR-200a regulates Nrf2 activation by targeting Keap1 mRNA in breast cancer cells. J. Biol. Chem..

[78] Li N., Muthusamy S., Liang R., Sarojini H., Wang E. (2011). Increased expression of miR-34a and miR-93 in rat liver during aging, and their impact on the expression of Mgst1 and Sirt1. Mech. Ageing Dev..

[79] Sangokoya C., Telen M.J., Chi J.-T. (2010). microRNA miR-144 modulates oxidative stress tolerance and associates with anemia severity in sickle cell disease. Blood J. Am. Soc. Hematol..

[80] Yang M., Yao Y., Eades G., Zhang Y., Zhou Q. (2011). MiR-28 regulates Nrf2 expression through a Keap1-independent mechanism. Breast Cancer Res. Treat..

[81] Li W., Liu H., Zhou J.-S., Cao J.-F., Zhou X.-B., Choi A.M., Chen Z.-H., Shen H.-H. (2012). Caveolin-1 inhibits expression of antioxidant enzymes through direct interaction with nuclear erythroid 2 p45-related factor-2 (Nrf2). J. Biol. Chem..

[82] Kaspar J.W., Niture S.K., Jaiswal A.K. (2009). Nrf2: INrf2 (Keap1) signaling in oxidative stress. Free Radic. Biol. Med..

[83] Houstis N., Rosen E.D., Lander E.S. (2006). Reactive oxygen species have a causal role in multiple forms of insulin resistance. Nature.

[84] Vasileva L.V., Savova M.S., Amirova K.M., Dinkova-Kostova A.T., Georgiev M.I. (2020). Obesity and NRF2-mediated cytoprotection: Where is the missing link?. Pharmacol. Res..

[85] Sarutipaiboon I., Settasatian N., Komanasin N., Kukongwiriyapan U., Sawanyawisuth K., Intharaphet P., Senthong V., Settasatian C. (2020). Association of genetic variations in NRF2, NQO1, HMOX1, and MT with severity of coronary artery disease and related risk factors. Cardiovasc. Toxicol..

[86] Cheng L., Jin Z., Zhao R., Ren K., Deng C., Yu S. (2015). Resveratrol attenuates inflammation and oxidative stress induced by myocardial ischemia-reperfusion injury: Role of Nrf2/ARE pathway. Int. J. Clin. Exp. Med..

[87] Amaral J.H., Rizzi E.S., Alves-Lopes R., Pinheiro L.C., Tostes R.C., Tanus-Santos J.E. (2019). Antioxidant and antihypertensive responses to oral nitrite involves activation of the Nrf2 pathway. Free Radic. Biol. Med..

[88] Delgobo M., Gonçalves R.M., Delazeri M.A., Falchetti M., Zandoná A., das Neves R.N., Almeida K., Fagundes A.C., Gelain D.P., Fracasso J.I. (2021). Thioredoxin reductase-1 levels are associated with NRF2 pathway activation and tumor recurrence in non-small cell lung cancer. Free Radic. Biol. Med..

[89] Suvorova E.S., Lucas O., Weisend C.M., Rollins M.F., Merrill G.F., Capecchi M.R., Schmidt E.E. (2009). Cytoprotective Nrf2 pathway is induced in chronically txnrd 1-deficient hepatocytes. PLoS ONE.

[90] Raninga P.V., Di Trapani G., Vuckovic S., Tonissen K.F. (2016). Cross-talk between two antioxidants, thioredoxin reductase and heme oxygenase-1, and therapeutic implications for multiple myeloma. Redox Biol..

[91] Aydemir D., Hashemkhani M., Durmusoglu E.G., Acar H.Y., Ulusu N.N. (2019). A new substrate for glutathione reductase: Glutathione coated Ag2S quantum dots. Talanta.

[92] Zhu H., Itoh K., Yamamoto M., Zweier J.L., Li Y. (2005). Role of Nrf2 signaling in regulation of antioxidants and phase 2 enzymes in cardiac fibroblasts: Protection against reactive oxygen and nitrogen species-induced cell injury. FEBS Lett..

[93] Dinić S., Grdović N., Uskoković A., Đorđević M., Mihailović M., Jovanović J.A., Poznanović G., Vidaković M. (2016). CXCL12 protects pancreatic β-cells from oxidative stress by a Nrf2-induced increase in catalase expression and activity. Proc. Jpn. Acad. Ser. B.

[94] Sadi G., Bozan D., Yildiz H.B. (2014). Redox regulation of antioxidant enzymes: Post-translational modulation of catalase and glutathione peroxidase activity by resveratrol in diabetic rat liver. Mol. Cell. Biochem..

[95] Mani M., Khaghani S., Mohammadi T.G., Zamani Z., Azadmanesh K., Meshkani R., Pasalar P., Mostafavi E. (2013). Activation of Nrf2-antioxidant response element mediated glutamate cysteine ligase expression in hepatoma cell line by homocysteine. Hepa. Mon..

[96] Solano-Urrusquieta A., Morales-González J.A., Castro-Narro G.E., Cerda-Reyes E., Flores-Rangel P.D., Fierros-Oceguera R. (2020). NRF-2 and nonalcoholic fatty liver disease. Ann. Hepatol..

[97] Rungratanawanich W., Abate G., Serafini M., Guarienti M., Catanzaro M., Marziano M., Memo M., Lanni C., Uberti D. (2018). Characterization of the antioxidant effects of γ-oryzanol: Involvement of the Nrf2 pathway. Oxid. Med. Cell. Longev..

[98] Bartolini D., Commodi J., Piroddi M., Incipini L., Sancineto L., Santi C., Galli F. (2015). Glutathione S-transferase pi expression regulates the Nrf2-dependent response to hormetic diselenides. Free Radic. Biol. Med..

[99] Nicolussi A., D’inzeo S., Capalbo C., Giannini G., Coppa A. (2017). The role of peroxiredoxins in cancer. Mol. Clin. Oncol..

[100] Kumar G., Mittal S., Sak K., Tuli H.S. (2016). Molecular mechanisms underlying chemopreventive potential of curcumin: Current challenges and future perspectives. Life Sci..

[101] Chang A., Jeske L., Ulbrich S., Hofmann J., Koblitz J., Schomburg I., Neumann-Schaal M., Jahn D., Schomburg D. (2021). BRENDA, the ELIXIR core data resource in 2021: New developments and updates. Nucleic Acids Res..

[102] Zhou Y., Duan S., Zhou Y., Yu S., Wu J., Wu X., Zhao J., Zhao Y. (2015). Sulfiredoxin-1 attenuates oxidative stress via Nrf2/ARE pathway and 2-Cys Prdxs after oxygen-glucose deprivation in astrocytes. J. Mol. Neurosci..

[103] Nguyen T., Sherratt P.J., Pickett C.B. (2003). Regulatory mechanisms controlling gene expression mediated by the antioxidant response element. Annu. Rev. Pharmacol. Toxicol..

[104] Chun K.-S., Raut P.K., Kim D.-H., Surh Y.-J. (2021). Role of chemopreventive phytochemicals in NRF2-mediated redox homeostasis in humans. Free Radic. Biol. Med..

[105] Gozzelino R., Jeney V., Soares M.P. (2010). Mechanisms of Cell Protection by Heme Oxygenase-1. Annu. Rev. Pharmacol. Toxicol..

[106] Soriano F.X., Léveillé F., Papadia S., Higgins L.G., Varley J., Baxter P., Hayes J.D., Hardingham G.E. (2008). Induction of sulfiredoxin expression and reduction of peroxiredoxin hyperoxidation by the neuroprotective Nrf2 activator 3H-1,2-dithiole-3-thione. J. Neurochem..

[107] Waza A.A., Hamid Z., Ali S., Bhat S.A., Bhat M.A. (2018). A review on heme oxygenase-1 induction: Is it a necessary evil. Inflamm. Res..

[108] Abraham N.G., Junge J.M., Drummond G.S. (2016). Translational Significance of Heme Oxygenase in Obesity and Metabolic Syndrome. Trends Pharmacol. Sci..

[109] Ross D., Siegel D. (2021). The diverse functionality of NQO1 and its roles in redox control. Redox Biol..

[110] Ross D., Siegel D. (2018). NQO1 in protection against oxidative stress. Curr. Opin. Toxicol..

[111] Samimi F., Baazm M., Eftekhar E., Rajabi S., Goodarzi M.T., Jalali Mashayekhi F. (2019). Possible antioxidant mechanism of coenzyme Q10 in diabetes: Impact on Sirt1/Nrf2 signaling pathways. Res. Pharm Sci.

[112] Rosa A.C., Bruni N., Meineri G., Corsi D., Cavi N., Gastaldi D., Dosio F. (2021). Strategies to expand the therapeutic potential of superoxide dismutase by exploiting delivery approaches. Int. J. Biol. Macromol..

[113] Ighodaro O.M., Akinloye O.A. (2018). First line defence antioxidants-superoxide dismutase (SOD), catalase (CAT) and glutathione peroxidase (GPX): Their fundamental role in the entire antioxidant defence grid. Alex. J. Med..

[114] Fujita H., Fujishima H., Chida S., Takahashi K., Qi Z., Kanetsuna Y., Breyer M.D., Harris R.C., Yamada Y., Takahashi T. (2009). Reduction of Renal Superoxide Dismutase in Progressive Diabetic Nephropathy. J. Am. Soc. Nephrol..

[115] Macmillan-Crow L.A., Cruthirds D.L. (2001). Manganese superoxide dismutase in disease. Free Radic. Res..

[116] Miao L., St.Clair D.K. (2009). Regulation of superoxide dismutase genes: Implications in disease. Free Radic. Biol. Med..

[117] Madsen-Bouterse S.A., Zhong Q., Mohammad G., Ho Y.-S., Kowluru R.A. (2010). Oxidative damage of mitochondrial DNA in diabetes and its protection by manganese superoxide dismutase. Free Radic. Res..

[118] Bartolini D., Giustarini D., Pietrella D., Rossi R., Galli F. (2020). Glutathione S-transferase P influences the Nrf2-dependent response of cellular thiols to seleno-compounds. Cell Biol. Toxicol..

[119] Kim C.H. (2013). Expression of extracellular superoxide dismutase protein in diabetes. Arch. Plast. Surg..

[120] Yamakura F., Kawasaki H. (2010). Post-translational modifications of superoxide dismutase. Biochim. Biophys. Acta Proteins Proteom..

[121] Holmgren A., Lu J. (2010). Thioredoxin and thioredoxin reductase: Current research with special reference to human disease. Biochem. Biophys. Res. Commun..

[122] Arnér E.S. (2009). Focus on mammalian thioredoxin reductases—important selenoproteins with versatile functions. Biochim. Biophys. Acta Gen. Subj..

[123] Stancill J.S., Happ J.T., Broniowska K.A., Hogg N., Corbett J.A. (2020). Peroxiredoxin 1 plays a primary role in protecting pancreatic β-cells from hydrogen peroxide and peroxynitrite. Am. J. Physiol. Regul. Integr. Comp. Physiol..

[124] Cesaratto L., Vascotto C., Calligaris S., Tell G. (2004). The importance of redox state in liver damage. Ann. Hepatol..

[125] Lillig C.H., Holmgren A. (2007). Thioredoxin and related molecules–from biology to health and disease. Antioxid. Redox Signal..

[126] Sinenko S.A., Starkova T.Y., Kuzmin A.A., Tomilin A.N. (2021). Physiological signaling functions of reactive oxygen species in stem cells: From flies to man. Front. Cell Dev. Biol..

[127] Singh C.K., Chhabra G., Ndiaye M.A., Garcia-Peterson L.M., Mack N.J., Ahmad N. (2018). The role of sirtuins in antioxidant and redox signaling. Antioxid. Redox Signal..

[128] Brigelius-Flohé R., Flohé L. (2020). Regulatory phenomena in the glutathione peroxidase superfamily. Antioxid. Redox Signal..

[129] Cebula M., Schmidt E.E., Arnér E.S.J. (2015). TrxR1 as a potent regulator of the Nrf2-Keap1 response system. Antioxid. Redox Signal..

[130] Dunigan K., Li Q., Li R., Locy M.L., Wall S., Tipple T.E. (2018). The thioredoxin reductase inhibitor auranofin induces heme oxygenase-1 in lung epithelial cells via Nrf2-dependent mechanisms. Am. J. Physiol-Lung Cell. Mol. Physiol..

[131] Dinkova-Kostova A.T., Abramov A.Y. (2015). The emerging role of Nrf2 in mitochondrial function. Free Radic. Biol. Med..

[132] Carlberg I., Mannervik B. (1985). Glutathione reductase. Methods Enzymol..

[133] Yang M., Chan H., Yu L. (2006). Glutathione peroxidase and glutathione reductase activities are partially responsible for determining the susceptibility of cells to oxidative stress. Toxicology.

[134] Lu S.C. (2009). Regulation of glutathione synthesis. Mol. Asp. Med..

[135] Vargas M.R., Johnson J.A. (2009). The Nrf2–ARE cytoprotective pathway in astrocytes. Expert Rev. Mol. Med..

[136] Kensler T.W., Wakabayashi N., Biswal S. (2007). Cell survival responses to environmental stresses via the Keap1-Nrf2-ARE pathway. Annu. Rev. Pharmacol. Toxicol..

[137] Singh A., Rangasamy T., Thimmulappa R.K., Lee H., Osburn W.O., Brigelius-Flohé R., Kensler T.W., Yamamoto M., Biswal S. (2006). Glutathione peroxidase 2, the major cigarette smoke–inducible isoform of GPX in lungs, is regulated by Nrf2. Am. J. Respir. Cell Mol. Biol..

[138] Sasaki H., Sato H., Kuriyama-Matsumura K., Sato K., Maebara K., Wang H., Tamba M., Itoh K., Yamamoto M., Bannai S. (2002). Electrophile response element-mediated induction of the cystine/glutamate exchange transporter gene expression. J. Biol. Chem..

[139] Rangasamy T., Cho C.Y., Thimmulappa R.K., Zhen L., Srisuma S.S., Kensler T.W., Yamamoto M., Petrache I., Tuder R.M., Biswal S. (2004). Genetic ablation of Nrf2 enhances susceptibility to cigarette smoke–induced emphysema in mice. J. Clin. Investig..

[140] Thimmulappa R.K., Lee H., Rangasamy T., Reddy S.P., Yamamoto M., Kensler T.W., Biswal S. (2016). Nrf2 is a critical regulator of the innate immune response and survival during experimental sepsis. J. Clin. Investig..

[141] Harvey C., Thimmulappa R., Singh A., Blake D., Ling G., Wakabayashi N., Fujii J., Myers A., Biswal S. (2009). Nrf2-regulated glutathione recycling independent of biosynthesis is critical for cell survival during oxidative stress. Free Radic. Biol. Med..

[142] Mofidi-Najjar F., Ghadari R., Yousefi R., Safari N., Sheikhhasani V., Sheibani N., Moosavi-Movahedi A.A. (2017). Studies to reveal the nature of interactions between catalase and curcumin using computational methods and optical techniques. Int. J. Biol. Macromol..

[143] Eremin A., Moroz I., Mikhailova R. (2008). Use of cadmium hydroxide gel for isolation of extracellular catalases from Penicillium piceum and characterization of purified enzymes. Appl. Biochem. Microbiol..

[144] Góth L., Nagy T. (2012). Acatalasemia and diabetes mellitus. Arch. Biochem. Biophys..

[145] Góth L., Lenkey Á., Bigler W.N. (2001). Blood Catalase Deficiency and Diabetes in Hungary. Diabetes Care.

[146] Lee D.-Y., Song M.-Y., Kim E.-H. (2021). Role of Oxidative Stress and Nrf2/KEAP1 Signaling in Colorectal Cancer: Mechanisms and Therapeutic Perspectives with Phytochemicals. Antioxidants.

[147] Chang S.-Y., Chen Y.-W., Zhao X.-P., Chenier I., Tran S., Sauvé A., Ingelfinger J.R., Zhang S.-L. (2012). Catalase prevents maternal diabetes–induced perinatal programming via the Nrf2–HO-1 defense system. Diabetes.

[148] Ma Q. (2013). Role of nrf2 in oxidative stress and toxicity. Annu. Rev. Pharmacol. Toxicol..

[149] Abdo S., Shi Y., Otoukesh A., Ghosh A., Lo C.-S., Chenier I., Filep J.G., Ingelfinger J.R., Zhang S.L., Chan J.S. (2014). Catalase overexpression prevents nuclear factor erythroid 2–related factor 2 stimulation of renal angiotensinogen gene expression, hypertension, and kidney injury in diabetic mice. Diabetes.

[150] Glorieux C., Zamocky M., Sandoval J.M., Verrax J., Calderon P.B. (2015). Regulation of catalase expression in healthy and cancerous cells. Free Radic. Biol. Med..

[151] Wheaton W.W., Chandel N.S. (2011). Hypoxia. 2. Hypoxia regulates cellular metabolism. Am. J. Physiol.-Cell Physiol..

[152] Kaluz S., Kaluzová M., Stanbridge E.J. (2008). Regulation of gene expression by hypoxia: Integration of the HIF-transduced hypoxic signal at the hypoxia-responsive element. Clin. Chim. Acta.

[153] Janke K., Brockmeier U., Kuhlmann K., Eisenacher M., Nolde J., Meyer H.E., Mairbäurl H., Metzen E. (2013). Factor inhibiting HIF-1 (FIH-1) modulates protein interactions of apoptosis-stimulating p53 binding protein 2 (ASPP2). J. Cell Sci..

[154] McNEILL L.A., Hewitson K.S., Claridge T.D., Seibel J.F., Horsfall L.E., Schofield C.J. (2002). Hypoxia-inducible factor asparaginyl hydroxylase (FIH-1) catalyses hydroxylation at the β-carbon of asparagine-803. Biochem. J..

[155] Semenza G.L. (2011). Oxygen sensing, homeostasis, and disease. N. Engl. J. Med..

[156] Panieri E., Saso L. (2019). Potential applications of NRF2 inhibitors in cancer therapy. Oxidative Med. Cell. Longev..

[157] Jiménez-Osorio A.S., Gonzalez-Reyes S., Pedraza-Chaverri J. (2015). Natural Nrf2 activators in diabetes. Clin. Chim. Acta.

[158] Ramsey C.P., Glass C.A., Montgomery M.B., Lindl K.A., Ritson G.P., Chia L.A., Hamilton R.L., Chu C.T., Jordan-Sciutto K.L. (2007). Expression of Nrf2 in neurodegenerative diseases. J. Neuropathol. Exp. Neurol..

[159] Lu Y., Wang B., Shi Q., Wang X., Wang D., Zhu L. (2016). Brusatol inhibits HIF-1 signaling pathway and suppresses glucose uptake under hypoxic conditions in HCT116 cells. Sci. Rep..

[160] Thum T., Galuppo P., Wolf C., Fiedler J., Kneitz S., van Laake L.W., Doevendans P.A., Mummery C.L., Borlak J.R., Haverich A. (2007). MicroRNAs in the human heart: A clue to fetal gene reprogramming in heart failure. Circulation.

[161] Lee S., Hallis S.P., Jung K.-A., Ryu D., Kwak M.-K. (2019). Impairment of HIF-1α-mediated metabolic adaption by NRF2-silencing in breast cancer cells. Redox Biol..

[162] Oh E.-T., Kim J.-w., Kim J.M., Kim S.J., Lee J.-S., Hong S.-S., Goodwin J., Ruthenborg R.J., Jung M.G., Lee H.-J. (2016). NQO1 inhibits proteasome-mediated degradation of HIF-1α. Nat. Commun..

[163] Thangarajah H., Yao D., Chang E.I., Shi Y., Jazayeri L., Vial I.N., Galiano R.D., Du X.-L., Grogan R., Galvez M.G. (2009). The molecular basis for impaired hypoxia-induced VEGF expression in diabetic tissues. Proc. Natl. Acad. Sci. USA.

[164] Ceradini D.J., Kulkarni A.R., Callaghan M.J., Tepper O.M., Bastidas N., Kleinman M.E., Capla J.M., Galiano R.D., Levine J.P., Gurtner G.C. (2004). Progenitor cell trafficking is regulated by hypoxic gradients through HIF-1 induction of SDF-1. Nat. Med..

[165] Ceradini D.J., Yao D., Grogan R.H., Callaghan M.J., Edelstein D., Brownlee M., Gurtner G.C. (2008). Decreasing intracellular superoxide corrects defective ischemia-induced new vessel formation in diabetic mice. J. Biol. Chem..

[166] Duh E., Aiello L.P. (1999). Vascular endothelial growth factor and diabetes: The agonist versus antagonist paradox. Diabetes.

[167] Manuelli V., Pecorari C., Filomeni G., Zito E. (2021). Regulation of redox signaling in HIF-1-dependent tumor angiogenesis. FEBS J..

[168] Satoh H., Moriguchi T., Saigusa D., Baird L., Yu L., Rokutan H., Igarashi K., Ebina M., Shibata T., Yamamoto M. (2016). NRF2 intensifies host defense systems to prevent lung carcinogenesis, but after tumor initiation accelerates malignant cell growth. Cancer Res..

[169] Toth R.K., Warfel N.A. (2017). Strange bedfellows: Nuclear factor, erythroid 2-like 2 (Nrf2) and hypoxia-inducible factor 1 (HIF-1) in tumor hypoxia. Antioxidants.

[170] Williams T.I., Lynn B.C., Markesbery W.R., Lovell M.A. (2006). Increased levels of 4-hydroxynonenal and acrolein, neurotoxic markers of lipid peroxidation, in the brain in Mild Cognitive Impairment and early Alzheimer’s disease. Neurobiol. Aging.

[171] Singh A., Kukreti R., Saso L., Kukreti S. (2019). Oxidative stress: A key modulator in neurodegenerative diseases. Molecules.

[172] Murakami K., Irie K., Ohigashi H., Hara H., Nagao M., Shimizu T., Shirasawa T. (2005). Formation and stabilization model of the 42-mer Aβ radical: Implications for the long-lasting oxidative stress in Alzheimer’s disease. J. Am. Chem. Soc..

[173] Keller J., Schmitt F., Scheff S., Ding Q., Chen Q., Butterfield D., Markesbery W. (2005). Evidence of increased oxidative damage in subjects with mild cognitive impairment. Neurology.

[174] Persson T., Popescu B.O., Cedazo-Minguez A. (2014). Oxidative stress in Alzheimer’s disease: Why did antioxidant therapy fail?. Oxid. Med. Cell. Longev..

[175] ALTamimi J.Z., AlFaris N.A., Al-Farga A.M., Alshammari G.M., BinMowyna M.N., Yahya M.A. (2021). Curcumin reverses diabetic nephropathy in streptozotocin-induced diabetes in rats by inhibition of PKCβ/p66Shc axis and activation of FOXO-3a. J. Nutr. Biochem..

[176] Amalraj A., Pius A., Gopi S., Gopi S. (2017). Biological activities of curcuminoids, other biomolecules from turmeric and their derivatives–A review. J. Tradit. Complement. Med..

[177] Hassan F.-U., Rehman M.S.-U., Khan M.S., Ali M.A., Javed A., Nawaz A., Yang C. (2019). Curcumin as an alternative epigenetic modulator: Mechanism of action and potential effects. Front. Genet..

[178] Wang H., Zhang K., Liu J., Yang J., Tian Y., Yang C., Li Y., Shao M., Su W., Song N. (2021). Curcumin Regulates Cancer Progression: Focus on ncRNAs and Molecular Signaling Pathways. Front. Oncol..

[179] Cho J.W., Lee K.S., Kim C.W. (2007). Curcumin attenuates the expression of IL-1beta, IL-6, and TNF-alpha as well as cyclin E in TNF-alpha-treated HaCaT cells; NF-kappaB and MAPKs as potential upstream targets. Int. J. Mol. Med..

[180] Sahebkar A., Cicero A.F.G., Simental-Mendía L.E., Aggarwal B.B., Gupta S.C. (2016). Curcumin downregulates human tumor necrosis factor-α levels: A systematic review and meta-analysis ofrandomized controlled trials. Pharmacol. Res..

[181] Yahfoufi N., Alsadi N., Jambi M., Matar C. (2018). The Immunomodulatory and Anti-Inflammatory Role of Polyphenols. Nutrients.

[182] Palizgir M.T., Akhtari M., Mahmoudi M., Mostafaei S., Rezaiemanesh A., Shahram F. (2018). Curcumin reduces the expression of interleukin 1β and the production of interleukin 6 and tumor necrosis factor alpha by M1 macrophages from patients with Behcet’s disease. Immunopharmacol. Immunotoxicol..

[183] Taghavi F., Rahban M., Moosavi-Movahedi A.A., Moosavi-Movahedi A.A. (2021). Lifestyle in the Regulation of Diabetic Disorders. Rationality and Scientific Lifestyle for Health.

[184] World Health Organization (2016). Global Report on Diabetes.

[185] Brownlee M., Cerami A., Vlassara H. (1988). Advanced glycosylation end products in tissue and the biochemical basis of diabetic complications. N. Engl. J. Med..

[186] Schmidt A.M., Hori O., Brett J., Yan S.D., Wautier J.-L., Stern D. (1994). Cellular receptors for advanced glycation end products. Implications for induction of oxidant stress and cellular dysfunction in the pathogenesis of vascular lesions. Arterioscler. Thromb. J. Vasc. Biol..

[187] Schmidt A.M., Hori O., Chen J.X., Li J.F., Crandall J., Zhang J., Cao R., Yan S., Brett J., Stern D. (1995). Advanced glycation endproducts interacting with their endothelial receptor induce expression of vascular cell adhesion molecule-1 (VCAM-1) in cultured human endothelial cells and in mice. A potential mechanism for the accelerated vasculopathy of diabetes. J. Clin. Investig..

[188] Vlassara H. (1996). Advanced glycation end-products and atherosclerosis. Ann. Med..

[189] Boudina S., Abel E.D. (2007). Diabetic cardiomyopathy revisited. Circulation.

[190] Lam C.S. (2015). Diabetic cardiomyopathy: An expression of stage B heart failure with preserved ejection fraction. Diabetes Vasc. Dis. Res..

[191] Soetikno V., Sari F.R., Veeraveedu P.T., Thandavarayan R.A., Harima M., Sukumaran V., Lakshmanan A.P., Suzuki K., Kawachi H., Watanabe K. (2011). Curcumin ameliorates macrophage infiltration by inhibiting NF-κB activation and proinflammatory cytokines in streptozotocin induced-diabetic nephropathy. Nutr. Metab..

[192] Jain S.K., Rains J., Croad J., Larson B., Jones K. (2009). Curcumin supplementation lowers TNF-α, IL-6, IL-8, and MCP-1 secretion in high glucose-treated cultured monocytes and blood levels of TNF-α, IL-6, MCP-1, glucose, and glycosylated hemoglobin in diabetic rats. Antioxid. Redox Signal..

[193] Yu W., Wu J., Cai F., Xiang J., Zha W., Fan D., Guo S., Ming Z., Liu C. (2012). Curcumin alleviates diabetic cardiomyopathy in experimental diabetic rats. PLoS ONE.

[194] Abo-Salem O.M., Harisa G.I., Ali T.M., El-Sayed E.S.M., Abou-Elnour F.M. (2014). Curcumin Ameliorates Streptozotocin-Induced Heart Injury in Rats. J. Biochem. Mol. Toxicol..

[195] Soetikno V., Sari F.R., Sukumaran V., Lakshmanan A.P., Mito S., Harima M., Thandavarayan R.A., Suzuki K., Nagata M., Takagi R. (2012). Curcumin prevents diabetic cardiomyopathy in streptozotocin-induced diabetic rats: Possible involvement of PKC–MAPK signaling pathway. Eur. J. Pharm. Sci..

[196] Abdel Aziz M.T., El-Asmar M.F., El Nadi E.G., Wassef M.A., Ahmed H.H., Rashed L.A., Obaia E.M., Sabry D., Hassouna A.A., Abdel Aziz A.T. (2010). The effect of curcumin on insulin release in rat-isolated pancreatic islets. Angiology.

[197] Aziz M.T.A., El Ibrashy I.N., Mikhailidis D.P., Rezq A.M., Wassef M.A.A., Fouad H.H., Ahmed H.H., Sabry D.A., Shawky H.M., Hussein R.E. (2013). Signaling mechanisms of a water soluble curcumin derivative in experimental type 1 diabetes with cardiomyopathy. Diabetol. Metab. Syndr..

[198] Yu W., Zha W., Ke Z., Min Q., Li C., Sun H., Liu C. (2016). Curcumin protects neonatal rat cardiomyocytes against high glucose-induced apoptosis via PI3K/Akt signalling pathway. J. Diabetes Res..

[199] Liu Y., Wang Y., Miao X., Zhou S., Tan Y., Liang G., Zheng Y., Liu Q., Sun J., Cai L. (2014). Inhibition of JNK by compound C66 prevents pathological changes of the aorta in STZ-induced diabetes. J. Cell. Mol. Med..

[200] Frank R. (2004). retinopathy D. Diabet. Retin. N. Engl. J. Med..

[201] Yau J.W., Rogers S.L., Kawasaki R., Lamoureux E.L., Kowalski J.W., Bek T., Chen S.-J., Dekker J.M., Fletcher A., Grauslund J. (2012). Global prevalence and major risk factors of diabetic retinopathy. Diabetes Care.

[202] Kowluru R. (2001). Diabetes-induced elevations in retinal oxidative stress, protein kinase C and nitric oxide are interrelated. Acta Diabetol..

[203] Brownlee M. (2005). The pathobiology of diabetic complications: A unifying mechanism. Diabetes.

[204] Lorenzi M. (2007). The polyol pathway as a mechanism for diabetic retinopathy: Attractive, elusive, and resilient. Exp. Diabetes Res..

[205] Inoguchi T., Battan R., Handler E., Sportsman J.R., Heath W., King G.L. (1992). Preferential elevation of protein kinase C isoform beta II and diacylglycerol levels in the aorta and heart of diabetic rats: Differential reversibility to glycemic control by islet cell transplantation. Proc. Natl. Acad. Sci. USA.

[206] Zhong Q., Mishra M., Kowluru R.A. (2013). Transcription factor Nrf2-mediated antioxidant defense system in the development of diabetic retinopathy. Investig. Ophthalmol. Vis. Sci..

[207] Kern T.S., Kowluru R.A., Engerman R.L. (1994). Abnormalities of retinal metabolism in diabetes or galactosemia: ATPases and glutathione. Investig. Ophthalmol. Vis. Sci..

[208] Mishra M., Zhong Q., Kowluru R.A. (2014). Epigenetic modifications of Nrf2-mediated glutamate–cysteine ligase: Implications for the development of diabetic retinopathy and the metabolic memory phenomenon associated with its continued progression. Free Radic. Biol. Med..

[209] Khor T.O., Huang Y., Wu T.-Y., Shu L., Lee J., Kong A.-N.T. (2011). Pharmacodynamics of curcumin as DNA hypomethylation agent in restoring the expression of Nrf2 via promoter CpGs demethylation. Biochem. Pharmacol..

[210] Bardak H., Uğuz A.C., Bardak Y. (2018). Protective effects of melatonin and memantine in human retinal pigment epithelium (ARPE-19) cells against 2-ethylpyridine-induced oxidative stress: Implications for age-related macular degeneration. Cutan. Ocul. Toxicol..

[211] Park S.-I., Lee E.H., Kim S.R., Jang Y.P. (2017). Anti-apoptotic effects of Curcuma longa L. extract and its curcuminoids against blue light-induced cytotoxicity in A2E-laden human retinal pigment epithelial cells. J. Pharm. Pharmacol..

[212] Woo J.M., Shin D.-Y., Lee S.J., Joe Y., Zheng M., Yim J.H., Callaway Z., Chung H.T. (2012). Curcumin protects retinal pigment epithelial cells against oxidative stress via induction of heme oxygenase-1 expression and reduction of reactive oxygen. Mol. Vis..

[213] Mathew T., Sarada S. (2018). Intonation of Nrf2 and Hif1-α pathway by curcumin prophylaxis: A potential strategy to augment survival signaling under hypoxia. Respir. Physiol. Neurobiol..

[214] Muhammad I., Wang X., Li S., Li R., Zhang X. (2018). Curcumin confers hepatoprotection against AFB 1-induced toxicity via activating autophagy and ameliorating inflammation involving Nrf2/HO-1 signaling pathway. Mol. Biol. Rep..

[215] Bucolo C., Drago F., Maisto R., Romano G.L., D’Agata V., Maugeri G., Giunta S. (2019). Curcumin prevents high glucose damage in retinal pigment epithelial cells through ERK1/2-mediated activation of the Nrf2/HO-1 pathway. J. Cell. Physiol..

[216] Kume S., Koya D., Uzu T., Maegawa H. (2014). Role of nutrient-sensing signals in the pathogenesis of diabetic nephropathy. Biomed. Res. Int..

[217] Gross J.L., De Azevedo M.J., Silveiro S.P., Canani L.H., Caramori M.L., Zelmanovitz T. (2005). Diabetic nephropathy: Diagnosis, prevention, and treatment. Diabetes Care.

[218] Zheng H., Whitman S.A., Wu W., Wondrak G.T., Wong P.K., Fang D., Zhang D.D. (2011). Therapeutic potential of Nrf2 activators in streptozotocin-induced diabetic nephropathy. Diabetes.

[219] Kim B.H., Lee E.S., Choi R., Nawaboot J., Lee M.Y., Lee E.Y., Kim H.S., Chung C.H. (2016). Protective effects of curcumin on renal oxidative stress and lipid metabolism in a rat model of type 2 diabetic nephropathy. Yonsei Med. J..

[220] Soetikno V., Sari F.R., Lakshmanan A.P., Arumugam S., Harima M., Suzuki K., Kawachi H., Watanabe K. (2013). Curcumin alleviates oxidative stress, inflammation, and renal fibrosis in remnant kidney through the N rf2–keap1 pathway. Mol. Nutr. Food Res..

[221] Soetikno V., Watanabe K., Sari F.R., Harima M., Thandavarayan R.A., Veeraveedu P.T., Arozal W., Sukumaran V., Lakshmanan A.P., Arumugam S. (2011). Curcumin attenuates diabetic nephropathy by inhibiting PKC-α and PKC-β1 activity in streptozotocin-induced type I diabetic rats. Mol. Nutr. Food Res..

[222] Balogun E., Hoque M., Gong P., Killeen E., Green C.J., Foresti R., Alam J., Motterlini R. (2003). Curcumin activates the haem oxygenase-1 gene via regulation of Nrf2 and the antioxidant-responsive element. Biochem. J..

[223] Zingg J.M., Hasan S.T., Meydani M. (2013). Molecular mechanisms of hypolipidemic effects of curcumin. Biofactors.

[224] Ghosh S.S., Massey H.D., Krieg R., Fazelbhoy Z.A., Ghosh S., Sica D.A., Fakhry I., Gehr T.W. (2009). Curcumin ameliorates renal failure in 5/6 nephrectomized rats: Role of inflammation. Am. J. Physiol.-Ren. Physiol..

[225] Lu M., Yin N., Liu W., Cui X., Chen S., Wang E. (2017). Curcumin ameliorates diabetic nephropathy by suppressing NLRP3 inflammasome signaling. Biomed. Res. Int..

[226] Tanase D.M., Gosav E.M., Neculae E., Costea C.F., Ciocoiu M., Hurjui L.L., Tarniceriu C.C., Maranduca M.A., Lacatusu C.M., Floria M. (2020). Role of gut microbiota on onset and progression of microvascular complications of type 2 diabetes (T2DM). Nutrients.

[227] Cameron N.E., Cotter M.A. (2008). Pro-inflammatory mechanisms in diabetic neuropathy: Focus on the nuclear factor kappa B pathway. Curr. Drug Targets.

[228] Mattson M.P., Camandola S. (2001). NF-κB in neuronal plasticity and neurodegenerative disorders. J. Clin. Investig..

[229] Okamoto K., Martin D.P., Schmelzer J.D., Mitsui Y., Low P.A. (2001). Pro-and anti-inflammatory cytokine gene expression in rat sciatic nerve chronic constriction injury model of neuropathic pain. Exp. Neurol..

[230] Vincent A.M., Brownlee M., Russell J.W. (2002). Oxidative stress and programmed cell death in diabetic neuropathy. Ann. N. Y. Acad. Sci..

[231] Patel S., Santani D. (2009). Role of NF-κB in the pathogenesis of diabetes and its associated complications. Pharmacol. Rep..

[232] Sharma S., Kulkarni S.K., Agrewala J.N., Chopra K. (2006). Curcumin attenuates thermal hyperalgesia in a diabetic mouse model of neuropathic pain. Eur. J. Pharmacol..

[233] Sharma S., Chopra K., Kulkarni S.K. (2007). Effect of insulin and its combination with resveratrol or curcumin in attenuation of diabetic neuropathic pain: Participation of nitric oxide and TNF-alpha. Phytother. Res..

[234] Daugherty D.J., Marquez A., Calcutt N.A., Schubert D. (2018). A novel curcumin derivative for the treatment of diabetic neuropathy. Neuropharmacology.

[235] Zhao W.-C., Zhang B., Liao M.-J., Zhang W.-X., He W.-Y., Wang H.-B., Yang C.-X. (2014). Curcumin ameliorated diabetic neuropathy partially by inhibition of NADPH oxidase mediating oxidative stress in the spinal cord. Neurosci. Lett..

[236] Oz M., Atalik K.E.N., Yerlikaya F.H., Demir E.A. (2015). Curcumin alleviates cisplatin-induced learning and memory impairments. Neurobiol. Learn. Mem..

[237] Zhang X., Guan Z., Wang X., Sun D., Wang D., Li Y., Pei B., Ye M., Xu J., Yue X. (2020). Curcumin alleviates oxaliplatin-induced peripheral neuropathic pain through inhibiting oxidative stress-mediated activation of NF-κB and mitigating inflammation. Biol. Pharm. Bulle.

